# Tumor heterogeneity and immune-evasive T follicular cell lymphoma phenotypes at single-cell resolution

**DOI:** 10.1038/s41375-023-02093-7

**Published:** 2023-11-27

**Authors:** Sakurako Suma, Yasuhito Suehara, Manabu Fujisawa, Yoshiaki Abe, Keiichiro Hattori, Kenichi Makishima, Tatsuhiro Sakamoto, Aya Sawa, Hiroko Bando, Daisuke Kaji, Takeshi Sugio, Koji Kato, Koichi Akashi, Kosei Matsue, Joaquim Carreras, Naoya Nakamura, Ayako Suzuki, Yutaka Suzuki, Ken Ito, Hiroyuki Shiiba, Shigeru Chiba, Mamiko Sakata-Yanagimoto

**Affiliations:** 1 Department of Hematology, University of Tsukuba Hospital, Tsukuba, Japan; 2 Department of Hematology, Institute of Medicine, University of Tsukuba, Tsukuba, Japan; 3 Centre for Lymphoid Cancer, BC Cancer, Vancouver, BC Canada; 4 Department of Breast-Thyroid-Endocrine Surgery, University of Tsukuba Hospital, Tsukuba, Japan; 5 Department of Hematology, Toranomon Hospital, Tokyo, Japan; 6 Department of Medicine, Division of Oncology, Stanford University, Stanford, CA USA; 7 Department of Medicine and Biosystemic Science, Kyushu University Graduate School of Medical Science, Fukuoka, Japan; 8 Division of Hematology/Oncology, Department of Internal Medicine, Kameda Medical Center, Kamogawa, Japan; 9 Department of Pathology, Tokai University School of Medicine, Isehara, Japan; 10 Department of Computational Biology and Medical Sciences, the University of Tokyo, Kashiwa, Japan; 11 Oncology Business Unit, Eisai Co., Ltd., Tsukuba, Japan; 12 Oncology Department, Medical Head Quarters, Eisai Co., Ltd., Tokyo, Japan; 13 Division of Advanced Hemato-Oncology, Transborder Medical Research Center, University of Tsukuba, Tsukuba, Japan

**Keywords:** T-cell lymphoma, Cancer microenvironment, Cancer therapeutic resistance, Cancer genomics

## Abstract

T follicular helper (T_FH_) cell lymphomas (TFHLs) are characterized by T_FH_-like properties and accompanied by substantial immune-cell infiltration into tumor tissues. Nevertheless, the comprehensive understanding of tumor-cell heterogeneity and immune profiles of TFHL remains elusive. To address this, we conducted single-cell transcriptomic analysis on 9 lymph node (LN) and 16 peripheral blood (PB) samples from TFHL patients. Tumor cells were divided into 5 distinct subclusters, with significant heterogeneity observed in the expression levels of T_FH_ markers. Copy number variation (CNV) and trajectory analyses indicated that the accumulation of CNVs, together with gene mutations, may drive the clonal evolution of tumor cells towards T_FH_-like and cell proliferation phenotypes. Additionally, we identified a novel tumor-cell-specific marker, PLS3. Notably, we found a significant increase in exhausted CD8^+^ T cells with oligoclonal expansion in TFHL LNs and PB, along with distinctive immune evasion characteristics exhibited by infiltrating regulatory T, myeloid, B, and natural killer cells. Finally, in-silico and spatial cell-cell interaction analyses revealed complex networking between tumor and immune cells, driving the formation of an immunosuppressive microenvironment. These findings highlight the remarkable tumor-cell heterogeneity and immunoevasion in TFHL beyond previous expectations, suggesting potential roles in treatment resistance.

## Introduction

Nodal T follicular helper cell (T_FH_) lymphomas (TFHLs) represent a distinct subtype of peripheral T-cell lymphomas (PTCLs), characterized by tumor cells exhibiting T_FH_-like properties [1–3]. Physiologically, T_FH_ cells interact with B cells through CD40 ligand (CD40LG)-CD40 signaling, as well as by secreting chemokines and cytokines, such as CXCL13, interleukin (IL)-21, and IL-4, to support B cell proliferation and differentiation in the germinal center of follicles [4]. In TFHL, malignant T cells also express CXCL13 and CD40LG [1, 5], and patients show high serum levels of cytokines, including IL-4, IL-6, and IL-21 [6, 7]. Although this release of cytokines and chemokines may lead to the prominent infiltration into TFHL tissue by immune cells [1], the details are unknown.

TFHLs carry a poor prognosis, with a 5-year overall survival of 30–40% and chemotherapy resistance [8]. While tumor-cell heterogeneity contributes to therapeutic resistance in various cancers [9], it remains incompletely understood in TFHL. Molecularly, genomic analysis has revealed that the *RHOA* G17V mutation (G17V) controls T_FH_ cell lineage differentiation, promoting the development of TFHL [10–12]. Moreover, G17V mutants acquire VAV1 binding capability, leading to activation of the T-cell receptor (TCR) signaling [10]. However, factors driving further tumor evolution and heterogeneity after tumor initiation remain poorly elucidated.

Generally, the tumor microenvironment (TME) controls immune evasion to support growth and metastasis [13], with high expression levels of immunosuppressive signatures within the TME associated with a poor TFHL prognosis [6, 7, 14, 15]. Although mounting evidence of this nodal interface indicates a primary role in tumor growth control, comprehensive immune profiling of the TFHL microenvironment remains incomplete.

Therefore, we aimed to profile the cell types and tumor cell characteristics involved in immune evasion and therapeutic resistance in TFHL by single-cell transcriptomic analysis.

## Methods

Detailed methods are described in Supplementary Methods.

### Human samples

**Fig. 1:**
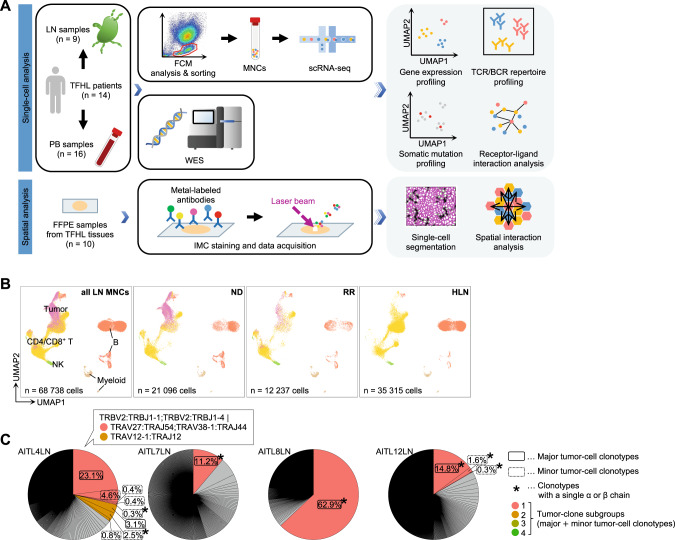
Transcriptomic profile of T follicular helper cell lymphoma (TFHL) samples. **A** Study overview and workflow of analysis. BCR B cell receptor, scRNA-seq, single-cell RNA sequencing, FCM flow cytometry, FFPE formalin-fixed paraffin-embedded, IMC imaging mass cytometry, LN lymph node, MNC mononuclear cell, PB peripheral blood, TCR T-cell receptor, UMAP Uniform Manifold Approximation and Projection for Dimension Reduction, WES whole-exome sequencing, **B** UMAP plots of all MNCs from nine TFHL LNs and seven homeostatic lymph nodes (HLNs), colored by cell type (left). Cells are shown separately for each clinical status (middle two and right). B, B cell; CD4/CD8^+^ T, CD4^+^ and CD8^+^ T cell; Myeloid, myeloid cell; NK, natural killer cell; ND newly diagnosed, TFHL; RR relapsed or refractory TFHL, Tumor, tumor cell. **C** Representative pie charts of TCR clonotypes for each sample. Tumor clones are colored.

This study was approved by the Institutional Review Board of the University of Tsukuba Hospital (Tsukuba, Japan) and other participating institutions and conducted in accordance with the Declaration of Helsinki. Written, informed consent was obtained from all participating patients. Prospectively, nine lymph node (LN; six from newly diagnosed [ND] patients and three from relapsed or refractory [RR] patients) and 16 peripheral blood (PB; including sequential samples from two patients) samples were collected from 14 TFHL patients (Fig. 1A; Table S1). Diagnoses were performed by expert hematopathologists at each institution. Seven homeostatic LNs (HLNs) from patients with non-hematologic cancers were collected for comparison.

### Flow cytometry (FCM) and single-cell library construction

Mononuclear cells (MNCs) from LN and PB samples were sorted for 5′ single-cell RNA (scRNA)- and T-/B-cell receptor (scTCR/BCR)-sequencing (seq) (Fig. S1A). Additionally, T cell-enriched scRNA/TCR-seq libraries were constructed from TFHL LN CD4/CD8^+^ T cells when sufficient cells were available (Fig. S1B; Table S1). Single-cell libraries were constructed using the Chromium system (10x Genomics, Pleasanton, CA, USA). Publicly available 5′ scRNA/TCR/BCR-seq data of 5 PB samples from healthy donors [16] were used as controls. HLN samples were verified as malignancy-free by pan-cytokeratin negativity (Fig. S1C). TFHL tumor cells were detected as a PD1^bright^ CD4^+^ T-cell population by FCM, as previously reported [17] (Fig. S1D).

### Genomic mutation detection using whole-exome sequencing (WES)

WES was performed on genomic DNA extracted from LNs, PB, and buccal swabs of the 14 TFHL patients (Table S1). Somatic mutations were described by the Genomon2 pipeline (v.2.6.2, https://github.com/Genomon-Project) as previously described [18, 19] with minor modifications. Germline mutations were excluded by buccal swab comparison. Somatic copy number variations (CNVs) were called using the Genome Analysis Tool Kit (v4.2) [20].

### Spatial analysis using imaging mass cytometry (IMC)

Spatial interaction analysis was performed on 10 formalin-fixed, paraffin-embedded samples from TFHL LNs using the Hyperion Imaging System (Standard Bio Tools Inc., South San Francisco, CA, USA) (Table S2). Cell-cell interactions were evaluated from cell-type spatial distributions [21].

### Statistical analysis

All statistical analyses were performed using R (v3.6.0, v3.6.2, or v4.0.2) on RStudio (v1.2.1578 or v1.2.5019). The Wilcoxon rank-sum test or MAST (for single-cell data only, v1.12.0) [22] was used to analyze differences between the two groups. Two-tailed statistical tests were performed, and *P* < 0.05 was considered statistically significant.

## Results

### Single-cell transcriptomic and TCR repertoire profiles of TFHL

We first analyzed scRNA-seq data of 68,738 LN and 98,891 PB cells collected from 14 TFHL patients and controls (Fig. 1A; Table S1). Unsupervised clustering and annotation by canonical markers revealed four main cell types: CD4/CD8^+^ T, natural killer (NK; NK/gamma-delta [γδ] T for PB), B, and myeloid cells (Fig. 1B, Fig. S2A).

Subsequently, we analyzed LN scTCR-seq data to identify tumor cells and found unique TCR clonotypes in 93.7% of T cells (Fig. S2B, C). Major and minor tumor-cell clonotypes were defined based on clonality (Fig. 1C, Fig. S2D; Table S3). The median proportion of tumor cells in all LN MNCs was 14.1% (range 5.7–83.8%; Table S4). In 7 of 9 TFHL LNs, tumor cells were clones within a patient, suggesting single-TCR clonal expansion (Fig. 1C, Fig. S2D). Contrastingly, in AITL4, two clones with identical TCRβ chains and differing TCRα chains expanded (Fig. 1C; Table S3). Notably, 31.3% (2652 cells) of tumor cells expressed only one productive α or β chain, contrasting dogma that αβ T cells physiologically express a pair of productive α and β chains (Fig. 1C, Fig. S2D). In three TFHLs (AITL7, AITL8, and AITL12), the single-TCR clonotype was detected in most tumor cells (Fig. S2E). Moreover, in these TFHLs, FCM analysis revealed the expansion of abnormal CD4^+^ PD1^high^ T-cell populations lacking cell-surface expression of TCRα and β chain-interacting CD3ε [23], which was not observed in TFHLs with physiological paired-chain TCRs (Fig. S2F, G, and data not shown). Tumor cell infiltration was also identified in 12 of 16 PB samples (0.24–74.0% in PB MNCs; Table S4), five of which showed expansion of single-chain TCR tumor cells with CD3ε^-^ CD4^+^ PD1^high^ T-cell populations by FCM, consistent with the LN data (Fig. S2F and S3A–C).

### Genomic characterization of TFHL

We next analyzed somatic mutations using bulk WES data to construct genomic profiles of TFHL, finding 397 total somatic mutations in 14 TFHL patients. This included recurrent *TET2* mutations in 11 (78.6%), G17V in 6 (42.9%), *DNMT3A* in 6 (42.9%), and *IDH2* R172 in 2 (14.3%), comparable to previous reports [24–27] (Fig. S4A; Table S5). Furthermore, we analyzed CNVs and found that gain of chromosome (chr) 5 was most frequently observed (35.7%; 5/14), and gains of chr7/7q, 19, 21, or 22q were also detected in ≥2 of 14 cases (14.3%) with co-occurrence of chr5 and 21 gain in 1 case (7.1%), consistent with previous reports [28] (Fig. S4B).

### Inter-patient and intra-tumor TFHL heterogeneity

**Fig. 2:**
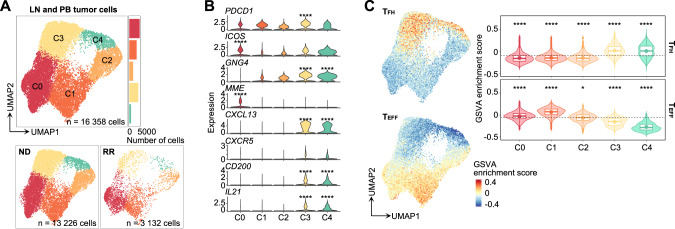
Single-cell analysis of tumor cells from TFHL LN and PB samples. **A** UMAP plots of LN and PB tumor cell subclusters (top). Cells are shown separately for each clinical status (bottom). **B** The expression levels of known T follicular helper (T_FH_) cell markers for each cluster. Adjusted *P* values are calculated by the Wilcoxon rank-sum test with Bonferroni correction across all genes expressed by tumor cells and shown only when there is a significant difference. **C** Feature (left panel) and violin (right panel) plots of gene set variation analysis (GSVA) enrichment score for T_FH_ (top) or CD4^+^ effector T cell (T_EFF_, bottom) signatures in tumor cells. In the violin plots, the dotted lines represent mean enrichment scores across all clusters. The boxplots show the median (center line), mean (center dot), interquartile range (box limits), and minimum to max values (whiskers) for each group. Adjusted *P* values are calculated by the pairwise Wilcoxon rank-sum test for each cluster against the mean value of all clusters. *P < 5.0 × 10^−2^, ****P < 1.0 × 10^−4^.

To investigate tumor cell characteristics and heterogeneity, we sorted 16,358 tumor cells from scRNA-seq data into five clusters by unsupervised clustering (C0–C4; Fig. 2A, Fig. S5A), finding consistent inter-patient heterogeneity (Fig. S5B). Notably, T_FH_ marker [4] expressions were highly heterogeneous between clusters (Fig. 2B, Fig. S5C; Table S6). *PDCD1* and *ICOS* were globally expressed, while *MME* (encoding CD10) and other T_FH_ markers were predominantly expressed in C0 and C3–4, respectively (Fig. 2B). Subsequently, we conducted gene set variation analysis (GSVA) [29] on each cell using T_FH_ and non-T_FH_ effector T cell (T_EFF_) signatures derived from bulk RNA-seq data (GSE58596 [30]). This revealed enrichment of the T_FH_ signature in C3–4 and the T_EFF_ signature in C0–2 (Fig. 2C). Moreover, cell-cycle scores revealed that C0, C1, and C3 were primarily in G1 stage, but C2 and C4 were exclusively in G2M or S stages, indicating active cell proliferation in C2 and C4 (Fig. S5D).

Next, we compared LN and PB tumor cells, revealing that C3–4 were predominant in LNs while C1–2 were more abundant in PB (Fig. S5E). Moreover, differentially expressed gene (DEG) analysis and GSVA revealed that LN tumor cells exhibited relatively higher expression of T_FH_ markers whereas PB tumor cells showed high expression of cytotoxicity-associated genes [31] (Fig. S5F, G). FCM analysis confirmed significantly higher PD1 expression in LN tumor cells versus those of PB (Fig. S5H, I). Subsequent trajectory analysis using Monocle3 [32] inferred that LN tumor cells terminally differentiated starting from C0 through C3 to C4, whereas PB tumor cells differentiated from C0 to C2 via C1 (Fig. S5J).

We next performed DEG and gene ontology (GO) analyses between tumor cells with single-chain and paired-chain TCRs (Fig. S5K). Unexpectedly, both types had activated TCR and cytokine signaling pathways (Fig. S5L). Notably, genomic mutations known to activate TCR pathways (G17V, *CTNNB1*), or those in TCR signaling-related genes (*LCK*, *KRAS*) [33], were detected in 4 of 5 cases with expanded single-chain TCR clonotypes (Table S5).

### Estimation of tumor evolution at single-cell resolution

**Fig. 3:**
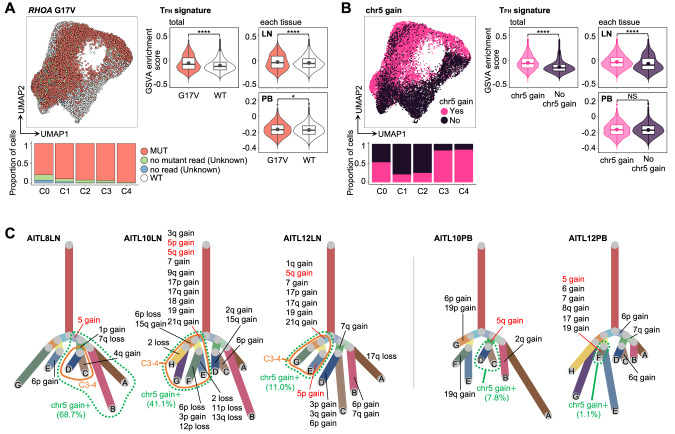
Estimation of genomic alternations at the single-cell level. **A** Distribution of *RHOA* G17V (G17V) mutant cells (left panel) and comparison of GSVA enrichment score for the T_FH_ signature between G17V mutant (red) and wild-type (WT, white) cells (right panel) in LN and PB tumor cells. In the violin plots of the right panel, adjusted *P* values are calculated for all tumor cells (left) and each tissue (right). “Unknown” cells had no mutant reads or no coverage. “WT” cells were from TFHL patients without G17V mutations by WES. MUT, mutant cells. **B** Distribution of tumor cells with chromosome (chr)5 gain (left panel) and comparison of GSVA enrichment score for the T_FH_ signature between tumor cells with chr5 gain (pink) and those without (blue) (right panel). In the violin plots of the right panel, adjusted *P* values are calculated for all tumor cells (left) and each tissue (right). NS, not significant. **C** Phylogenetic trees based on copy number variation (CNV) patterns identified by inferCNV [35] in TFHL LN (left panel) and PB (right panel) samples with partial chr5 gain. All boxplots show the median (center line), mean (center dot), interquartile range (box limits), and minimum to max values (whiskers) for each group. *P < 5.0 × 10^−2^, ****P < 1.0 × 10^−4^.

To assess mutational evolution using scRNA-seq tumor cell data, we reanalyzed the initially detected 11 somatic mutations and focused on mutations with a total read coverage ≥100×, identifying seven mutations present in over 20 cells, of which only G17V was detected in multiple patients (Fig. S6A–C; Table S7). In TFHL patients where G17V was initially identified by WES, G17V was detected in 86.7% of tumor cells while 9.4% of the cells lacked mutant reads and 3.9% had no coverage (Fig. 3A). Considering the frequent allelic dropout in scRNA-seq [34], cells without mutant reads or no coverage status were categorized as “unknown”. Conversely, cells from TFHL patients without G17V mutations by WES were dubbed “G17V wild-type (WT)” (Fig. 3A). GSVA, DEG, and GO analyses revealed enrichment of T_FH_ signatures and pathways related to the adaptive immune system and B-cell activation, including *CD40LG*, in G17V mutant cells compared to G17V WT cells, consistent with our previous report [18] (Fig. 3A, Fig. S6D, E). In AITL4LN/PB, with two distinctive tumor clones featuring identical TCRβ chains and different TCRα, *LRRC41* mutations were detected only in tumor-clone 1 (Fig. S6F). Based on UMAP visualization, these cells with different clones exhibited different gene expression profiles, indicating clonal evolution across divergent genetic/transcriptomic directions (Fig. S6G).

Next, we conducted single-cell level estimation of CNVs in LN tumor cells and classified them into subgroups based on their CNV patterns using inferCNV [35]. The estimated CNVs exhibited a high degree of consistency with paired bulk WES results (Fig. S4B and S7A). Similar to WES, the most frequently observed CNV in LN tumor cells was chr5 gain (6/9; 66.7%), comprising four samples detected by both scRNA-seq and WES, and two samples exclusively identified through scRNA-seq (AITL10LN and AITL12LN; Fig. S7A). In these two samples and AITL8LN, chr5 gain was identified only in a portion of tumor cells with the identical TCR clones, suggesting that it was acquired after TCR clonal expansion (Fig. S7A). Notably, chr5 gain was most prevalent in C3–4, showing enriched T_FH_ phenotypes (C3–4) and active cell proliferation (C4), followed by C0 (Fig. 3B). Consistently, DEG analysis revealed that T_FH_-related genes, including *CXCR5*, *CXCL13*, *IL21*, and *CD40LG*, were significantly upregulated in chr5-gain tumor cells compared to those without and GSVA showed that the T_FH_ signature enrichment was observed in total and LN tumor cells while being insignificant in PB tumor cells (Fig. 3B, Fig. S7B). Furthermore, the phylogenetic trees of partial chr5-gain samples diverged into two major branches based on the presence or absence of chr5 gain, plus the subsets with chr5 gain accompanied by other CNVs frequently detected in TFHL [28] (e.g., gains of chr7, 19, and 21) that evolved into C3–4 (Fig. 3C). In these samples, the T_FH_ signature was significantly enriched in chr5-gain positive cells compared to non-gain (Fig. S7C). Contrastingly, in AITL10PB and AITL12PB, where partial chr5 gain was detected in LN tumor cells, fewer chr5-gain cells were detected in PB tumor cells compared to those of LNs (41.1 versus 7.8% and 11.0 versus 1.1%), and most of the tumor cells belonged to C0–2, suggesting that C0–2 without chr5 gain and T_FH_ signatures favored infiltration into PB versus C3–4 (Fig. 3C, Fig. S7D). Indeed, CNV scores [36] were significantly higher in C3–4 and LN tumor cells than those in C0–2 and PB tumor cells, respectively, indicating the genomic complexity of C3–4 and LN tumor cells (Fig. S7E).

In summary, genomic aberrations, particularly G17V and chr5 gain, were closely correlated with distinct transcriptomic profiles in TFHL tumor cells at the single-cell resolution.

### PLS3 as a novel, tumor-specific marker

**Fig. 4:**
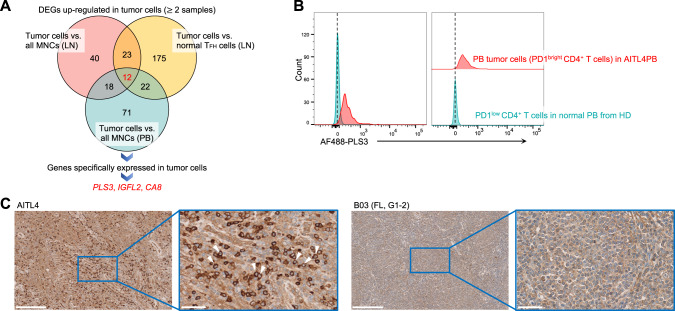
Identification of novel tumor-specific cell markers. **A** Overview of tumor-specific cell marker identification. For both LN (*n* = 9) and tumor cell-bearing PB (*n* = 12) samples, differentially expressed gene (DEG) analysis was performed to select genes whose expression was significantly elevated in tumor cells of two or more samples versus (vs.) all MNCs or normal T_FH_ cells (for only LN tumor cells) for all samples. Next, genes specifically expressed in tumor cells were selected by manual inspection using UMAP plots. **B** Representative FCM plots of PLS3 expression in PD1^bright^ CD4^+^ cells from PB of TFHL (AITL4, red) and PD1^dim^ CD4^+^ cells from PB of a healthy donor (HD, blue). **C** Representative images of PLS3 expression by immunohistochemical staining of FFPE samples from TFHL (AITL4, left) and B-cell lymphoma (B03, right). White triangles indicate tumor cells expressing PLS3 on the cell membrane surface. Images were scanned using the NanoZoomer (2.0HT, Hamamatsu Photonics, Shizuoka, Japan) and acquired at ×10 and ×40 with the NDP.view.2 software (v2.9.29). FL, follicular lymphoma; G, grade of FL according to the 4th edition of the World Health Organization classification [1]; Scale bar, 250 μm (left) and 50 μm (right).

To discover novel tumor-specific markers, we conducted DEG analysis using scRNA-seq data between LN/PB tumor cells and non-malignant MNCs or normal T_FH_ cells (for LNs only). DEGs with significantly higher expression in tumor cells across ≥2 samples, as well as those upregulated in both LN and PB tumor cells, were extracted (Fig. 4A; Table S8). Finally, *PLS3*, *IGFL2*, and *CA8* were identified as candidate genes (Fig. S8A; Table S9). FCM was performed to validate PLS3, revealing high expression in both LN and PB tumor cells versus non-malignant T cells (Fig. 4B). Furthermore, immunohistochemical staining revealed that PLS3 was strongly expressed on the tumor cell-surface in 17 of 35 PTCLs (10 of 20 angioimmunoblastic T-cell lymphomas, 2 of 5 nodal PTCLs with T_FH_ phenotypes, and 5 of 10 PTCLs not otherwise specified), accounting for 48.6% PTCL samples examined, while significant expression was observed in only one of 168 (0.6%) mature B-cell lymphomas (Fig. 4C; Table S10). These findings suggest that PLS3 is a promising, tumor-specific marker in PTCL.

### Dysfunctional CD8^+^ and regulatory T-cell expansion

**Fig. 5:**
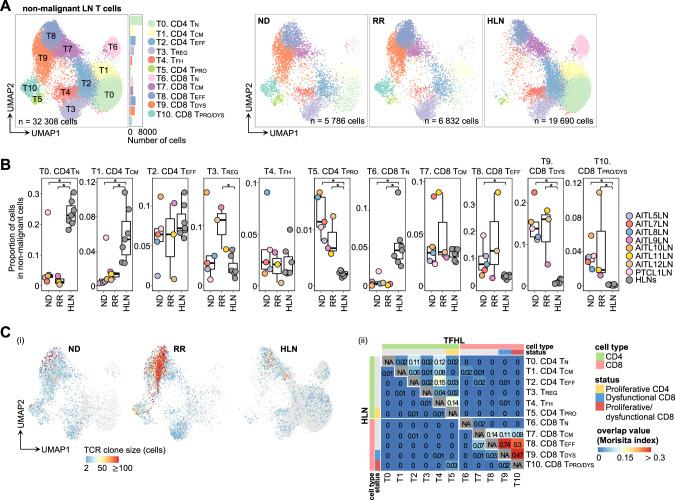
Subclustering of non-malignant T cells from LNs. **A** UMAP plots of non-malignant LN T cell subclusters. Cells are shown separately for each clinical status (right panels). T_CM_, central memory T cell; T_DYS_, dysfunctional T cell; T_EFF_, effector T cell; T_N_, naïve T cell; T_PRO_, proliferative T cell; T_PRO/DYS_, proliferative dysfunctional T cell; T_REG_, regulatory T cell. **B** Comparison of proportions of each cluster in non-malignant MNCs per sample. The boxplots show the median (center line), interquartile range (box limits), minimum to max values (whiskers), and samples (dots) for each group. *P* values are shown only for significant differences. *P < 5.0 × 10^−2^. **C** Clone size (i) and overlap analysis (ii) of TCRs in non-malignant LN T cells. The number of cells expressing each clonotype was defined as clone size and illustrated for each cell. The “repOverlap” function of Immunarch measured the TCR sharing between each cluster, analyzed and illustrated for TFHL LNs (upper right) and HLNs (lower left), respectively.

Subsequently, subclustering of non-malignant LN T cells highlighted 11 clusters (T0–10). Based on canonical markers, these clusters were classified as conventional CD4^+^ or CD8^+^ T, encompassing naïve (T_N_, T0 and T6), central memory (T_CM_, T1 and T7), T_EFF_ (T2 and T8), regulatory T (T_REG_, T3), T_FH_ (T4), proliferating CD4^+^ T (CD4 T_PRO_, T5), dysfunctional CD8^+^ T (CD8 T_DYS_, T9), and proliferating dysfunctional CD8^+^ T (CD8 T_PRO/DYS_, T10) (Fig. 5A, Fig. S9A–E). T9 and T10 expressed “exhaustion” or “dysfunctional” markers such as *PDCD1*, *HAVCR2* (encoding TIM-3), *TIGIT*, and *LAG3* [37, 38] (Fig. S9C, D). T5 was subclassified into two *FOXP3*^+^ *IL2RA*^+^ clusters (T5 FOXP3-1 and T5 FOXP3-2) and one cluster positive for the T_FH_ marker (T5 PDCD1) (Fig. S9F), suggesting that T5 was a mixture of proliferating T_REG_ and T_FH_. These manual annotations were further validated using SingleR [39, 40] (Fig. S9G).

The proportions of CD4 T_PRO_ and CD8 T_DYS_/T_PRO/DYS_ significantly increased in TFHL LNs versus HLNs, whereas those of CD4/CD8 T_N_ and CD4 T_CM_ decreased as previously reported [41] (Fig. 5B). Additionally, the proportion of T_REG_ was higher in RR TFHL LNs than HLNs (Fig. 5B, Fig. S9H). Moreover, compared with HLNs, T_REG_ in RR TFHL LNs exhibited higher expressions of genes associated with T_REG_ inhibitory functions, such as *FOXP3, IL2RA*, *TNFRSF4*, *TNFRSF9*, and *LAG3* [42], suggesting T_REG_ activation in RR TFHL (Fig. S9I; Table S11). Additionally, non-malignant PB T cell subclustering demonstrated the expansion of CD8 T_DYS_/T_PRO/DYS_ and upregulation of activated markers in T_REG_ from TFHL (Fig. S10A–F; Supplementary Note 1).

We next analyzed the TCR clonality of non-malignant LN T cells and identified oligo-clonal expansion of TCRs in CD8 T_EFF_, T_DYS_, and T_PRO/DYS_ of TFHL (Fig. 5C(i), Fig. S10G). Indicative of a common cellular origin, these cells partly shared identical TCRs between clusters, an overlap not observed in HLNs (Fig. 5C(ii)). Moreover, trajectory analysis revealed that a portion of CD8 T_EFF_ diverged and differentiated into CD8 T_DYS_ and T_PRO/DYS_ in TFHL, consistent with TCR analysis (Fig. S10H). Notably, TCR commonality was observed between LN and PB CD8 T_PRO/DYS_ of TFHL, suggesting that enrichment of immunoevasive phenotypes in PB potentially reflects the LN immune environment (Fig. S10I).

### Increased immunosuppressive myeloid cells

**Fig. 6:**
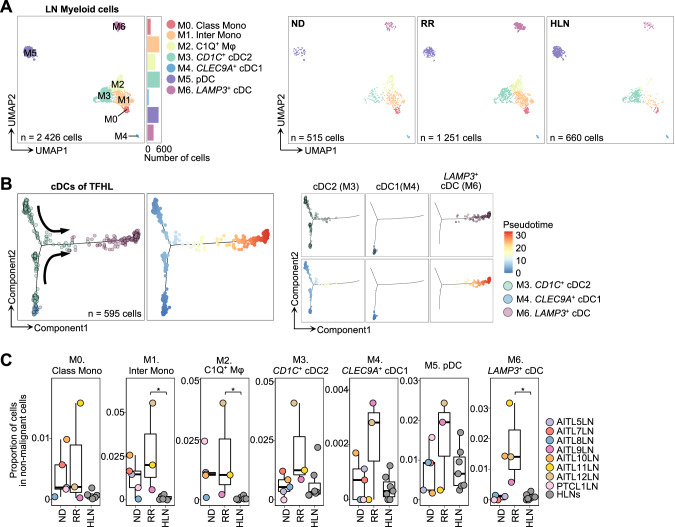
Subclustering of myeloid cells from LNs. **A** UMAP plots of LN myeloid cell subclusters. Cells are shown separately for each clinical status (right panels). C1Q^+^ Mφ, complement component C1q positive macrophage; CD1C^+^ cDC2, CD1C-positive type2 conventional dendritic cell; Class Mono, classical monocyte; *CLEC9A*^+^ cDC1, *CLEC9A*-positive type1 cDC; Inter Mono, intermediate monocyte; *LAMP3*^+^ cDC, *LAMP3*-positive cDC; pDC, plasmacytoid DC. **B** Trajectory inference by Monocle2 for cDCs of TFHL, color-coded by cluster (left of the left panel) and pseudo-time (right of the left panel). Cells are shown separately for each cluster (right panel). **C** Comparison of proportions of each cluster in non-malignant MNCs per sample. The boxplots show the median (center line), interquartile range (box limits), minimum to max values (whiskers), and samples (dots) for each group. *P* values are shown only when there is a significant difference. *P  < 5.0 × 10^−2^.

Subclustering LN myeloid cells distinguished seven clusters (M0–6; Fig. 6A). Employing canonical markers and SingleR analysis [43, 44], we annotated each cluster as classical and intermediate monocytes (Class Mono, M0; Inter Mono, M1, respectively), complement component 1Q (C1Q)-positive macrophages (C1Q^+^ Mφ, M2), *CD1C*-positive type 2 conventional dendritic cells (CD1C^+^ cDC2, M3), *CLEC9A*-positive type 1 cDCs (*CLEC9A*^+^ cDC1, M4), plasmacytoid DCs (pDC, M5), and *LAMP3*-positive cDCs (*LAMP3*^+^ cDC, M6) (Fig. 6A, Fig. S11A–D).

Notably, C1Q^+^ Mφ exhibited high C1Q-family gene expression (*C1QA*, *C1QB*, and *C1QC*), *CD163* expression, and ligands for T-cell immune checkpoints, including *LGALS9* (binds to TIM-3) and *NECTIN2* (binds to TIGIT) (Fig. S11C), resembling tumor-associated macrophages with immunosuppressive signatures reported in multiple cancers [43, 45]. Trajectory inference showed a continuous spectrum from Class/Inter Mono to C1Q^+^ Mφ (Fig. S11E). *LAMP3*^+^ cDCs were characterized by high immune regulatory gene expression, including *CD274* (encoding PD-L1) and *PDCD1LG2* (encoding PD-L2) [46], consistent with “mregDCs,” a subset of DCs co-expressing maturation and immunoregulatory genes identified in various cancers [43, 46] (Fig. S11B, C and F). *LAMP3*^+^ cDCs have been proposed to develop from either cDC1s or cDC2s [43, 46]. Using a previously reported scoring system [43, 46], we inferred that most *LAMP3*^+^ cDCs in TFHL originated from cDC2s, unlike many cancers [43, 46] (Fig. S11G). Trajectory analyses further supported this, revealing a continuous connection from cDC2 to *LAMP3*^+^ cDCs (Fig. 6B). Next, we compared the proportions of each cluster and observed a significant increase in Inter Mono, C1Q^+^ Mφ, and *LAMP3*^+^ cDCs in RR TFHL LNs than in HLNs (Fig. 6C). Finally, DEG analysis showed the expression level of *CCL17*, a ligand for *CCR4* [47], was significantly higher in *LAMP3*^+^ cDCs of RR TFHL LNs than HLNs (Fig. S11H, I).

Taken together, a distinct increase in immunoregulatory myeloid cells, particularly *LAMP3*^+^ cDCs, was observed in RR TFHL, which could support an immune-evasive TME.

### Exhausted phenotype and clonal expansion of B cells

**Fig. 7:**
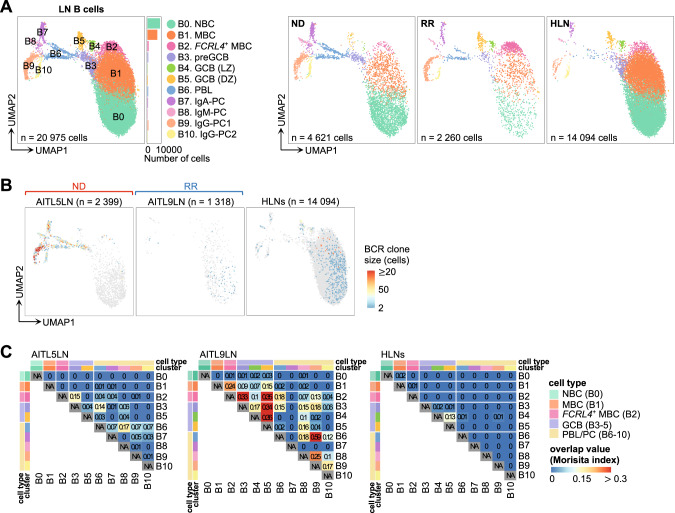
Subclustering of B cells from LNs. **A** UMAP plots of LN B cell subclusters. Cells are shown separately for each clinical status (right panels). *FCRL4*^+^ MBC, *FCRL4*-positive memory B cell; GCB (DZ), germinal center B cell in the dark zone; GCB (LZ), GCB in the light zone; MBC, memory B cell; NBC, naïve B cell; PBL, plasmablast; PC, plasma cell; preGCB, pre-germinal center B cell. **B** Clone size of BCRs in B cells from AITL5LN, AITL9LN, and HLNs. The number of cells expressing each clonotype was defined as clone size and calculated for each sample. HLN samples are shown as integrated (right). **C** BCR overlap analysis of B cells from AITL5LN, AITL9LN, and HLNs. AITL5LN and AITL9LN were analyzed individually while HLN samples were analyzed after integration.

Subclustering analysis of LN B cells revealed 11 clusters: naïve B cell (NBC, B0), memory B cell (MBC, B1), *FCRL4-*positive MBC (*FCRL4*^+^ MBC, B2), pre-germinal center B cell (preGCB, B3), light-zone GCB (GCB [LZ], B4), dark-zone GCB (GCB [DZ], B5), plasmablast (PBL, B6), and plasma cell (PC, B7–10) (Fig. 7A, Fig. S12A–C). *FCRL4*^+^ MBCs displayed “exhausted” or “atypical” MBC phenotypes, as observed in chronic inflammatory or immunodeficiency conditions [48–50], distinguished by high expression of *TBX21* (encoding T-bet), B cell inhibitory receptor genes (*FCRL4* and *SIGLEC6* [49]), and homing receptor genes (*CXCR3* and *ITGAX* [48, 49]) as well as low expression of *CR2* (encoding CD21) and *CD27* (Fig. S12D; Table S12). Compared to HLNs, the proportions of each cluster varied between TFHL patients (Fig. S12A). While statistically insignificant, the proportion of *FCRL4*^+^ MBCs was markedly increased (>20% of B cells) in 2 (AITL9LN and AITL12LN) out of 3 RR TFHLs (Fig. S12A). Moreover, DEG analysis highlighted the greater upregulation of genes related to exhausted MBC phenotypes [48–51] in *FCRL4*^+^ MBCs from RR TFHL LNs versus HLNs, although not significantly in ND TFHL (Fig. S12E; Table S13). Additionally, GO analysis and gene set enrichment analysis revealed the extensive enrichment of GCB- and CD40-related and AITL-specific GCB signatures [18] in broad B-cell clusters of TFHL, consistent with our previous findings [18] (Fig. S12F, G; Table S14).

Repertoire analysis was performed for five TFHL LNs (AITL5LN and AITL7–10LN), as well as all HLNs, revealing clonally expanded BCRs of GCBs and PBL/PCs in AITL5LN and AITL9LN (Fig. 7B, Fig. S12H–I). The BCRs of GCBs and PBL/PCs from AITL5LN and AITL9LN overlapped with *FCRL4*^+^ MBCs (Fig. 7C). Particularly in AITL9LN, trajectory analysis showed continuity of trajectories from GCBs to *FCRL4*^+^ MBCs and PBL/PCs among the cells sharing BCRs (Fig. S12J).

### NK-cell activation and exhaustion in RR TFHL

LN NK cell subclustering identified three clusters: proliferative NK (NK_PRO_, NK0), *XCL1*-positive tissue-resident NK [52] (*XCL1*^+^ NK, NK1), and *FCGR3A* (encoding CD16)-positive activated NK [53] (*FCGR3A*^+^ NK, NK2) (Fig. S13A–D). We found increases in the proportions of NK_PRO_ and *FCGR3A*^+^ NKs in TFHL LNs compared to all TFHL and HLNs (Fig. S13E). Moreover, DEG and GO analyses revealed proinflammatory cytokine pathway enhancement (e.g., interferon-γ) as well as reduced expression of a receptor-activating adapter molecule, *TYROBP* [54] (encoding DAP12), specifically in *XCL1*^+^ NK of TFHL LNs versus HLNs (Fig. S13F; Table S15, 16). Additionally, higher expressions of inhibitory genes, such as *HAVCR2* and *KLRC1* (encoding NKG2A) [53], were observed in *XCL1*^+^ NKs of RR TFHL but not in those of ND TFHL (Fig. S13G; Table S15, 16). These data suggest sustained activation of NK cells in TFHL LNs and subsequent elevation of inhibitory signaling, particularly in RR TFHL.

### In-silico and spatial intercellular interactions and formation of the TFHL immune-evasive microenvironment

To investigate the cellular interactions within the TME of TFHL LNs, we conducted in silico cell-cell network analysis using sc-RNAseq data through CellphoneDB [55] and NicheNetR [56] pipelines. The CellphoneDB analysis identified significant crosstalk among various cell types related to immunoregulation, including T_REG_, CD8 T_DYS_, *FCRL4*^+^ MBCs, C1Q^+^ Mφ, and tumor cells, which was more prominent in RR than ND TFHL (Fig. S14A). Moreover, the NicheNetR analysis revealed the potential ligands expressed by C3–4 of LN tumor cells that drive exhausted signatures of CD8 T_DYS_ in RR TFHL, including *IL21*, *TNF*, *TGFB1*, *CD80*, and *CD86* (Fig. S14B–D).

**Fig. 8:**
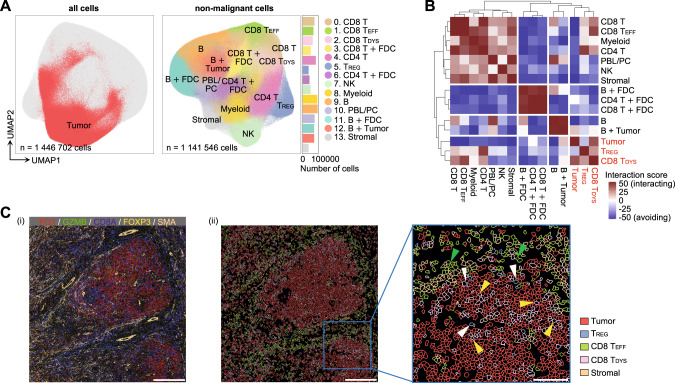
Cell-cell interaction analysis between tumor and immune cells from TFHL tissues. **A** UMAP plots of all (left) and non-malignant (right) cells detected by single-cell segmentation of IMC data from 10 FFPE samples of TFHL tissues. The tumor-cell cluster, identified by unsupervised clustering of all cells, is colored in red (left). The non-malignant cells are color-coded by subclusters identified by unsupervised clustering without the tumor-cell cluster (right). FDC, follicular dendritic cell; Stromal, stromal cell; +, a mixture of two cell types. **B** Spatial interaction analysis for each cell type using IMC data of TFHL tissues. Higher interaction scores indicate closer distance and more interaction between cells, whereas lower scores indicate farther distance and less interaction. **C** Representative IMC images (P5) color-coded by (i) expression levels of markers for CD8 T_DYS_ (PD1), CD8 T_EFF_ (GZMB), T_REG_ (FOXP3), and stromal cells (SMA) and (ii) cell types identified by clustering at the single-cell resolution. In panel (ii), only tumor cells, stromal cells, T_REG_ (white arrowheads), CD8 T_EFF_ (green arrowheads), and CD8 T_DYS_ (yellow arrowheads) are shown. Scale bar, 300 μm ((i) and left of (ii)) and 100 μm (right of (ii)).

To further validate the results of scRNA-seq data, we performed single-cell spatial analysis by IMC using 27 antibodies (Table S17) for samples from 10 TFHL patients (Table S2). Overall, 1,446,702 cells were identified by the single-cell segmentation process. Unsupervised clustering revealed expansion of a T_FH_-like tumor cell cluster (Fig. 8A, Fig. S15A, B). Subsequent clustering of non-malignant cells identified 14 subclusters, including GZMB^high^ PD1^low^ CD8 T_EFF_ (cluster 1), GZMB^low^ PD1^high^ CD8 T_DYS_ (cluster 2), and T_REG_ (cluster 5) (Fig. 8A, Fig. S15C, D; Table S18; Supplementary Note 2).

To assess cell-cell interactions [21], intercellular distances were calculated from the spatial distribution of each cell (Table S19). Tumor cells were significantly adjacent to CD8 T_DYS_ but distant from CD8 T_EFF_ and T_REG_ were adjacent to CD8 T_DYS_, suggesting potential cell-cell interactions within the TFHL TME (Fig. 8B–C).

## Discussion

Integration of single-cell and spatial analyses allowed us to comprehensively elucidate tumor cell profiles, as well as immunosuppressive and dysfunctional immune cells, revealing synergy in establishing an immunoevasive TME that co-evolves with disease progression.

Although increased exhausted CD8^+^ T cells and T_REG_s have been reported in TFHL [41], as well as solid cancers [37, 38], we found oligoclonal expansion and TCR commonality of CD8 T_DYS_ between LNs and PB for the first time in TFHL. Persistent antigenic stimulation and T_REG_ infiltration might lead to clonal expansion and augment exhaustion phenotypes of CD8^+^ T cells [38]. Clonal proliferation of B-lineage cells, as well as T-lineage tumor cells, is well reported for TFHL [1–3]. We previously reported that abnormal GCBs clonally expand under direct interaction with tumor cells [18]. In this study, we further identified that exhausted MBCs, resembling MBC subsets that regulate inappropriate immune responses in chronic infections [48, 49], also expanded with common clones with GCBs and PCs in TFHL. By employing single-cell analysis, we identified immunosuppressive *LAMP3*^+^ cDCs, likely promoting T_REG_ migration into tumor tissues through the CCL17-CCR4 axis [47], in addition to increased CD163-positive macrophages previously associated with adverse TFHL prognoses [7, 14].

Tumor cells may acquire T_FH_-like and cell proliferation phenotypes during clonal evolution through CNV accumulation, including chr5 gain, consistent with the previously reported increased expression of T-cell activation-associated genes, including *IL4*, in cases with chr5 gain [28]. Acquisition of T_FH_-like properties by tumor cells may modulate the TME to impart a self-survival advantage, driving self-activation and proliferation through interactions with immune cells [4]. As a limitation of tumor cell analysis in this study, AITL4PB accounts for the majority of PB tumor cells, which may lead to biased characteristics of PB tumor cells.

Intriguingly, the absence of CD3 surface expression in tumor cells, a known hallmark of TFHL [57], was associated with aberrant single-chain TCRs. Despite lacking a functional TCR-CD3 complex, these tumor cells exhibited upregulated TCR signaling pathways essential for T-cell development and survival [58] and frequent TCR-related gene mutations, indicating the acquisition of autonomous TCR signaling capacity during tumor progression without external antigen stimulation.

We identified *PLS3* as a tumor-specific marker through scRNA-seq and further confirmed its protein expression on the PTCL tumor cell-surface. PLS3, an actin-bundling protein, is physiologically expressed in cells within solid tissues such as fibroblasts and endothelial cells [59]. Notably, PLS3 is ectopically expressed in Sézary syndrome tumor cells, a leukemic variant of cutaneous T-cell lymphomas [60], but remains uninvestigated in other T-cell lymphomas. In our cohort of nodal PTCLs, approximately half expressed PLS3, with or without T_FH_ phenotypes. Moreover, we found that the expression profiles of known T_FH_ markers were very heterogeneous even within the same TFHL sample. PD1, a broadly positive marker for TFHL tumor cells, is also detectable in normal T_FH_ cells; combining PD1 with PLS3 may thus enhance diagnostic accuracy.

Overall, our study provides valuable insights into tumor-immune interactions and tumor cell characteristics mediated by genomic alterations which contribute to an immunoevasive environment in TFHL. This study may lead to the development of innovative therapeutic strategies targeting tumor immunity, ultimately addressing the challenge of therapeutic resistance in TFHL.

### Supplementary information

Supplementary information

Supplementary tables

## Data Availability

The scRNA-seq data are deposited in the European Genome-phenome Archive and available upon request. All other data are available from the corresponding author upon reasonable request (sakatama@md.tsukuba.ac.jp [MS-Y]).
