# Supplementary material for: Tumor heterogeneity and immune-evasive T follicular cell lymphoma phenotypes at single-cell resolution
Source: Leukemia. 2023 Nov 27;38(2):340–50. doi: 10.1038/s41375-023-02093-7 (PMC10844096; doi:10.1038/s41375-023-02093-7)
Supplement: Supplementary file 1 — Supplementary information [file 41375_2023_2093_MOESM1_ESM.pdf]

Supplemental Data

Tumor heterogeneity and immune-evasive T follicular cell lymphoma phenotypes at single-cell resolution

Sakurako Suma[1], Yasuhito Suehara[1], Manabu Fujisawa[2, 3], Yoshiaki Abe[2], Keiichiro Hattori[1, 2], Kenichi Makishima[1], Tatsuhiro Sakamoto[1, 2], Aya Sawa[4], Hiroko Bando[4], Daisuke Kaji[5], Takeshi Sugio[6], Koji Kato[7], Koichi Akashi[7], Kosei Matsue[8], Joaquim Carreras[9], Naoya Nakamura[9], Ayako Suzuki[10], Yutaka Suzuki[10], Ken Ito[11], Hiroyuki Shiiba[12], Shigeru Chiba[1, 2], and Mamiko Sakata-Yanagimoto[1, 2, 13]

[1] *Department of Hematology, University of Tsukuba Hospital, Tsukuba, Japan.*

[2] *Department of Hematology, Institute of Medicine, University of Tsukuba, Tsukuba, Japan.*

[3] *Centre for Lymphoid Cancer, BC Cancer, Vancouver, BC, Canada.*

[4] *Department of Breast-Thyroid-Endocrine Surgery, University of Tsukuba Hospital, Tsukuba, Japan.*

[5] *Department of Hematology, Toranomon Hospital, Tokyo, Japan.*

[6] *Department of Medicine, Division of Oncology, Stanford University, Stanford, CA, USA.*

[7] *Department of Medicine and Biosystemic Science, Kyushu University Graduate School of Medical Science, Fukuoka, Japan.*

[8] *Division of Hematology/Oncology, Department of Internal Medicine, Kameda Medical Center, Kamogawa, Japan.*

[9] *Department of Pathology, Tokai University School of Medicine, Isehara, Japan.*

[10] *Department of Computational Biology and Medical Sciences, the University of Tokyo, Kashiwa, Japan.*

[11] *Oncology Business Unit, Eisai Co., Ltd., Tsukuba, Japan.*

[12] *Oncology Department, Medical Head Quarters, Eisai Co., Ltd., Tokyo, Japan.*

[13] *Division of Advanced Hemato-Oncology, Transborder Medical Research Center, University of Tsukuba, Tsukuba, Japan.*

Supplementary Methods

Supplementary Notes

Supplementary Table Legends

Supplementary Figures (with legends)

Supplementary Methods

Patients and clinical sample collection

Lymph node (LN) and peripheral blood (PB) samples were collected from 14 patients with T follicular helper cell (T_{FH}) lymphomas (TFHLs) at the University of Tsukuba Hospital, Kameda Medical Center (Kamogawa, Japan), and Toranomon Hospital (Tokyo, Japan) between October of 2019 and February of 2021. For two of the TFHL patients (AITL1 and AITL2), PB samples were collected at two-time points: once upon new diagnosis (ND) ($AITL1_{ND}$ and $AITL2_{ND}$) and again upon refractory/relapsed (RR) disease ($AITL1_{RR}$ and $AITL2_{RR}$). LN samples from AITL5 and AITL7 were analyzed in a previously reported study [1]. For single-cell RNA sequencing (scRNA-seq) data from AITL4, only tumor cells were analyzed because systemic steroid administration for coexisting collagen disease may affect the composition of immune cells. Additionally, seven homeostatic LN (HLN) samples were also collected from patients with non-hematological malignancies at the University of Tsukuba Hospital and were confirmed as metastatic-free by flow cytometric (FCM) analysis of pan-cytokeratin. The patient characteristics are summarized in Table S1.

Sample processing

LN samples were minced immediately after excision and filtered with a 70-μm strainer. The cells were washed with phosphate-buffered saline (PBS, Nissui, Tokyo, Japan), containing 0.04% weight/volume bovine serum albumin (BSA, Sigma-Aldrich, Louis, MO, USA), and centrifuged twice for 5 min at 300 × *g* and 4°C. After removal of the supernatant, cells were resuspended in PBS/0.04% BSA and cell count and viability were estimated using a hemocytometer and trypan blue. After centrifugation for 5 min at 300 × *g* and 5°C once again,

cells were resuspended in PBS/0.04% BSA and diluted to 1×10^7 cells/ml in preparation for analysis or cryopreservation.

PB samples were stored at 4°C immediately after collection and processed within 24 h. After red blood cell (RBC) lysis by the ammonium-chloride-potassium buffer, cells were washed once with PBS/0.04% BSA and centrifuged for 5 min at 400 × *g* and 4°C. After removal of the supernatant, cells were resuspended in 20 ml of PBS/0.04% BSA buffer and cell count and viability were estimated. Cells were centrifuged for 5 min at 300 × *g* and 4°C, diluted to 1×10^7 cells/ml and kept on ice until use.

FCM analysis and sorting

Surface protein expression analysis and sorting for scRNA-seq were performed using cell suspensions obtained from the LN and PB samples described above. To prevent non-specific reactions mediated by Fc receptors (FcRs), 20 $\mu l/10^7$ cells of FcR Blocking Reagent (Miltenyi Biotec, Bergisch Gladbach, Germany) was added, and cell suspensions were incubated in a refrigerator for 10 min, followed by staining based on the recommended concentration of each antibody. Dead cells and doublets were removed by 7-amino-actinomycin D (7-AAD, Thermo Fisher Scientific, Waltham, MA, USA) staining and forward versus side scatter plots (Fig. S1A–B). FCM sorting and analysis were performed on BD FACSAria III (BD Biosciences, San Jose, CA, USA). FlowJo software (v10.7.1, Tree Star Inc., Ashland, OR, USA) and the Ggplot2 package (v3.3.3) [2] were used for FCM data analysis and visualization. All antibodies used are listed in Table S20.

Library preparation, sequencing, and pre-processing of single-cell data

The scRNA-seq libraries for mononuclear cells (MNCs) from LNs and PB were prepared using a Chromium Single Cell 5′ Library & Gel Bead Kit (v1 chemistry, 10x Genomics, Pleasanton, CA, USA) or a Chromium Next GEM Single Cell 5′ Library & Gel Bead Kit (v1.1 chemistry) according to the manufacturer's instructions (CG000086 Rev L or CG000207 Rev E), aiming for 5 000 cells per library. Simultaneously, single-cell T- and B-cell receptor sequencing (scTCR/BCR-seq) was performed for samples with sufficient T or B cells (> 20% of all LN MNCs or > 10% of all PB MNCs; Table S1 and S4). scTCR/BCR-seq libraries were constructed using a Chromium Single Cell V(D)J Reagent Kit (v1 or v1.1 chemistry). T cell-enriched scRNA/TCR-seq libraries were generated similarly to the MNCs using sorted CD4/CD8^{+} T cells. Quantification and quality control of libraries were performed using a 2100 Bioanalyzer System with High Sensitivity DNA Kit (Agilent Technologies, Santa Clara, CA, USA) and a KAPA Library Quantification Kit for Illumina platforms (KAPA Biosystems, Wilmington, MA, USA). Each cDNA library was sequenced on the HiSeq X system (Illumina, San Diego, CA, USA) with a paired-end 150-base read option. The publicly available 5′ scRNA-seq and scTCR/BCR-seq data from PB of 5 healthy donors (HDs) [3] generated by a Chromium system (Chromium Single Cell 5′ Reagent Kit v2 chemistry and Chromium Single Cell V(D)J Reagent Kit) were used as controls (Table S1). The age and sex of individual donors were obtained from Supplementary Figure S1 of the original paper [3].

Using the "cellranger count" function of the Cell Ranger pipeline (v3.1.0, 10x Genomics), we aligned scRNA-seq data to the reference genome (GRCh38) and performed preliminary filtering and counting unique molecular identifiers (UMIs) for downstream analysis. For scTCR/BCR-seq data, sequence assembly and paired clonotype calling were performed with the "cellranger vdj" function. scRNA-seq and scTCR/BCR-seq data, after implementing

the Cell Ranger pipeline, were primarily analyzed using R (v3.6.0, v3.6.2 or v4.0.2) on RStudio (v1.2.1578 or v1.2.5019).

Quality control (QC) of scRNA-seq data

First, to remove low-quality cells and genes, only genes expressed by more than three cells and cells expressing over 200 genes were included in further analyses. Next, we applied the Scater package (v1.14.6) [4] to filter out outliers from count matrices for each sample. Briefly, we used several common QC metrics to detect outliers: library size, which was defined as the total sum of counts across all relevant features for each cell; the number of expressed features in each cell; and the percentage of reads mapped to mitochondrial transcripts. Outliers were identified based on the median absolute deviation from the median value of each QC metric across all cells. Cells identified as outliers were considered to be low quality and discarded. After excluding mitochondrial and ribosomal genes, the Seurat package (v3.2.3) [5,6] was applied to log-normalize the count data by the default scale factor of 10 000.

Batch effects correction and data integration

The mutual nearest neighbors (MNN) batch correction technique in the Batchelor package (v1.2.4) [7] was utilized to remove batch effects between datasets resulting from technical biases and biological differences. Unlike the original method, the "batchelor::fastMNN" function performs principal components analysis (PCA) on the previously selected highly variable features (HVFs) to preemptively reduce the dimensions and speed up MNN identification and correction. For integrating data from LNs of patients with TFHL and HLNs, we used 2 000 HVFs and the first 50 principal components (PCs) for

downstream steps. For data from PB, 3 000 HVFs and the first 100 PCs were used instead. In LN data, variable genes of TCRαβ and immunoglobulins (*TRAV*, *TRBV*, *IGHV*, *IGKV*, and *IGLV*) were removed from the HVF selection to avoid their effect on clustering. We applied the "RunFastMNN" function in the SeuratWrappers package (v0.3.0; https://github.com/satijalab/seurat-wrappers) for running the "fastMNN" function with a Seurat object.

Unsupervised clustering and non-linear dimensional reduction

Subsequently, we applied Seurat to perform unsupervised graph-based clustering and Uniform Manifold Approximation and Projection (UMAP) visualization using the MNN-corrected components as input. Using the "FindNeighbors" function, a k-nearest neighbor graph was constructed based on the Euclidean distance and a shared nearest neighbor graph was generated by computing the neighborhood overlap between any two cells. Next, the "FindClusters" function was used to identify clusters by the Louvain algorithm (default setting), which was a shared nearest neighbor modularity optimization-based clustering algorithm. For visualization, UMAP transformation was performed using the "RunUMAP" function.

Finding cluster biomarkers and cell type annotation

To characterize each cluster, we used the "FindMarkers" or "FindAllMarkers" functions in the Seurat package to select upregulated genes in each cluster relative to other clusters. These functions performed the Wilcoxon Rank Sum test between pairs of clusters and a *P*-value adjustment using the Bonferroni correction. Clusters were manually assigned to a cell type by comparing the marker genes of each cluster and known canonical markers. Specifically, we

annotated CD3 gene (*CD3D*, *CD3E,* and *CD247*)- and *CD4* or CD8 gene (*CD8A* and *CD8B*)-positive clusters as "CD4/CD8^{+} T cells", *CD79A*-positive clusters as “B cells," and myeloid cell marker (*LYZ*, *CD68,* and *CST3*) [8,9]-positive clusters as "myeloid cells." CD3 gene-positive or weakly positive but *CD4* and CD8 gene-negative and natural killer (NK) cell marker (*XCL1*, *FCGR3A*, *KLRD1,* and *KLRF1*) [10]- or γδT cell marker (*TRDV2*, *TRGV9*, *TRDC*, *TRGC2,* and *TRGC1*) [11]-positive clusters were named as “NK cells” (for LNs) or “NK/γδT cells” (for PB samples). We also identified a *CD34*-positive "progenitor cell" cluster as a rare cell type (81 cells in all PB MNCs) in PB. Platelet clusters characterized by *PPBP*, *GP9,* and *PF4* and RBC clusters characterized by *HBB* and *HBA1* were excluded from downstream analyses. For clusters featured by the expression of multiple cell-type marker genes, we used the scDblFinder package (v1.4.0) [12] and confirmed that most cells in these clusters were doublets. These cells were also removed from further analysis.

Subsequently, after identifying tumor cells by the method described below, we subclustered immune cells by major cell types and annotated each cluster based on canonical markers. Results of manual annotations were confirmed by automated annotation by the SingleR package (v1.4.1) [13] with reference to previously published single-cell data.

Single-cell TCR/BCR repertoire analysis

We identified clonotypes from scTCR/BCR-seq data after running the Cell Ranger pipeline. Clonotypes were identified by sets of cells with unique and “productive” TCRα- and/or β-chains (for B cells, immunoglobulin [Ig] heavy [IgH] chains and two types of light [IgL] chains: κ or λ [IgK or IgL] chains), defined by identical complementarity-determining region 3 (CDR3) sequences. Here, a "productive" sequence refers to an mRNA sequence that could be translated into a functional protein. Those TCR- or Ig-chain sequences judged as productive met the

following conditions: were full length (include from the beginning of the V gene to the end of the J gene), started with a start codon, contained no stop codon, were in-frame, contained a CDR3 sequence, and matched the structural requirements calculated from the length of the V and J gene regions of the sequence (https://support.10xgenomics.com/single-cell-vdj/software/pipelines/3.1/algorithms/annotation). These clonotypes were named clonotype 1, clonotype 2, and so on from the most clonally extended ones for each sample. Generally, αβT and B cells express a pair of functional α- and β-chains or H- and L-chains but, occasionally, a single cell expresses multiple functional TCR or Ig chains [14–17]. Therefore, we detected up to two productive TCRα- and β-chains (IgH- and L-chains) in each cell. Downstream analysis of scTCR/BCR-seq was performed using the Immunarch package (v0.6.6) [18].

Tumor cell detection in TFHL LNs and PB

For LNs, we defined clonotypes that were expressed by over 20 $CD4^+$ T cells and accounted for over 1.5% of all T cells with TCRs as “major tumor-cell clonotypes” and cells with tumor-cell clonotypes as “tumor cells” (Fig. 1C; Table S3). Clonotypes composed only of the TCR α- and/or β-chains identical to the major tumor-cell clonotypes and expressed by five or more $CD4^+$ T cells were defined as “minor tumor-cell clonotypes.” Cells expressing a minor tumor-cell clonotype were also defined as tumor cells. Major and minor tumor-cell clnotypes with the same TCR chains were grouped together as “tumor-clone subgroups” (Table S3).

In nine PB samples, with their paired LNs also subjected to single-cell sequencing, cells harboring identical clonotypes to those of their respective LN tumor cells were defined as tumor cells (Fig. S16A). In the remaining seven PB samples without paired LN single-cell data, clonotypes that were expressed by over 100 cells and accounted for more than 10% of all TCRs were defined as tumor-cell clonotypes. In patients with TFHL with sequential samples (AITL1

and AITL2), cells expressing the clonotypes identical to those of tumor cells of the sequential sample were defined as tumor cells, even if no clonotype met the definition above (Fig. S16B).

Differentially expressed gene (DEG) analysis

We performed DEG analysis using the "FindMarkers" or "FindAllMarkers" functions of Seurat. The model-based analysis of single-cell transcriptomics (MAST, v1.12.0) [19] method was mainly used for DEG detection, and *P*-values were corrected by the Bonferroni method. In addition, the Wilcoxon rank sum test confirmed the DEGs detected by MAST. Genes with an adjusted *P*-value < 0.05 in both tests, with a log fold-change (logFC) of the average expression between the two groups ≥ 0.15 (logfc.threshold = 0.15), and expressed by $\geq 10\%$ of cells in the cluster (min.pct = 0.1) were considered significant. An adjusted *P*-value calculated by MAST was used to create a volcano plot. Metascape [20] and gene set enrichment analysis (GSEA; v4.1.0) [21] were used for gene ontology (GO) and pathway analyses of DEGs in specific clusters, respectively.

Gene set variation analysis (GSVA)

The GSVA package (v1.34.0) [22] was applied to estimate signatures or pathways enriched by each cell and cluster. The normalized data after log transformation was used as input, and the Gaussian kernel was selected as the kernel function used for the nonparametric estimation of enrichment scores for each cell. Gene sets for GSVA were downloaded from the Molecular Signatures Database (MSigDB; http://www.gsea-msigdb.org/gsea/msigdb/index.jsp) or extracted from a previously published RNA-seq dataset by DEG analysis using the edgeR package (v3.28.1) [23,24]. We used raw count data as input for DEG analysis by edgeR, filtered

out low-expression genes using the “filterByExpr” function, performed trimmed mean of M-values (TMM) normalization using the “calcNormFactors” function, and applied a generalized linear model (GLM) using the likelihood ratio test (LRT) with the “glmLRT” function. DEGs with an adjusted *P*-value < 0.05 and logFC ≥ 1 were extracted by the “decideTests” function. Finally, we performed the pairwise Wilcoxon test and Bonferroni *P*-value correction using the rstatix package (v0.7.0) [25] to compare median GSVA enrichment scores for each cluster.

Trajectory analysis

Trajectory analysis was performed using the Monocle2 (v2.18.0) [26], Monocle3 (v0.2.3.0) [27], and Slingshot (v1.8.0) [28] packages. The count data before normalization after QC was used as the input data for Monocle2. After estimating size factors and dispersions with the “estimateSizeFactors” and “estimateDispersions” functions, DEGs for each cluster were extracted with the “differentialGeneTest” function. Using only DEGs with a *P*-value < 0.01, the “reduceDimension” function performed a DDRTree reduction, and the “orderCells” function sorted the cells in trajectory order. In the Monocle3 analysis, we first converted the Seurat object to a "cell_data_set" object that could be used in Monocle3 by the “as.cell_data_set” function. After clustering with the “cluster_cells” function, trajectory estimation and cell sorting were performed using the “learn_graph” and “orderCells” functions, using the results of dimensional reduction by UMAP executed by Seurat. In trajectory analysis by Slingshot, after converting the Seurat object to a "SingleCellExperiment" object using the “as.SingleCellExperiment” function, dimensionality reduction data after batch effect correction by fastMNN and clustering results by Seurat were used. Trajectory estimation was performed by the “slingshot” function. We used the rgl package (v0.107.14, https://github.com/dmurdoch/rgl) for 3D plots.

Cell-cell interaction analysis

Ligand-receptor interactions between each cluster were estimated using the Python package CellPhoneDB (v3.0.0) [29] and the R package NicheNetR (v1.0.0) [30]. We used Python v3.8.5 for this analysis. In the CellPhoneDB analysis, we first performed the "statistical method" and obtained *P*-values. Specifically, only ligand-receptor pairs expressed by over 10% of the cells in each cluster were selected for analysis. Then, we randomly permuted the cluster labels of all cells 1 000 times to create a null distribution of the mean of the average ligand and receptor expression levels in the interacting clusters. *P*-values for the probability of enrichment within clusters for each ligand-receptor complex were obtained by calculating the percentage of the means equal to or higher than the actual mean. Subsequently, we performed the "DEGs analysis method," using DEGs upregulated in each cluster of LNs from patients with ND or RR TFHL versus HLNs as the input genes of the DEG list. As for CD8 T_{DYS} (T9), since the number of cells belonging to CD8 T_{DYS} of HLNs was too small to perform proper DEG analysis, we compared them with CD8 T_{EFF} (T8) of HLNs. Genes whose expression profiles were significantly elevated in the tumor cells versus non-malignant cells were used as the DEGs for the tumor cells. In the "DEGs analysis method," only ligand-receptor pairs expressed by over 10% of the cells in each cluster and for which at least one gene was included in the DEG list were extracted as significant interactions. Finally, interactions detected as significant by both methods were extracted and the numbers of interactions for each cluster were plotted in heatmaps.

NicheNetR is a tool for predicting ligands that can promote gene expression changes in specific target cells ("receiver/target"). In this analysis, the group of genes representing gene expression changes in target cells was defined as a "gene set of interest (geneset_oi)." The CD8

T_{DYS} (T9) cluster and tumor cells from RR TFHL were set as receivers and senders, respectively. Initially, genes expressed by over 10% cells in each cluster designated as a receiver or sender were used as "expressed_genes_receiver" and "expressed_genes_sender," respectively. Scince the number of cells belonging to CD8 T_{DYS} of HLNs was too small to perform proper DEG analysis, DEGs between CD8 T_{DYS} of RR TFHL LNs and CD8 T_{EFF} (T8) of HLNs with an adjusted P-value < 0.05, min.pct = 0.1, and logFC ≥ 0.35 were used as "geneset_oi." Next, we used the "predict_ligand_activities" function to estimate and rank the potential ligands of target genes in "geneset_oi." Among the potential ligands, the top 20 were used as the "best_upstream_ligands" for the following analysis. Furthermore, the "get_weighted_ligand_target_links" and "lr_network" functions predicted target genes and receptors of the "best_upstream_ligands." Finally, only bonafide interactions based on validated curated ligand-receptor databases were visualized in heatmaps.

Whole-exome sequencing (WES) and data processing

Genomic DNA were extracted from fresh frozen tumor tissue and PB samples, formalin-fixed paraffin-embedded (FFPE) tumor samples, and paired buccal swab samples to be used as reference using a QIAamp DNA Blood Mini Kit or GeneRead DNA FFPE Kit (Qiagen, Hilden, Germany) (Table S1). After DNA integrity number (DIN) values were measured using Agilent TapeStation 2200 (Agilent Technologies) for quality control, DNA was fragmented with DNA Shearing S220 (Covaris, Woburn, MA, USA). Exome capturing was performed using a SureSelect XT Human All Exon V7 kit (Agilent Technologies). Next, 150-bp paired-end sequencing was carried out according to the manufacturer's recommendations on a HiSeq X Ten system (Illumina).

Sequencing reads were aligned to the reference genome (GRCh37) using the Burrows-Wheeler Aligner (v.0.7.8). Mutation calling was performed using the Genomon2 pipeline (v.2.6.2, https://github.com/Genomon-Project) as previously described [1,31,32] with minor modifications. Putative somatic mutations with a Fisher's exact *P*-value < 0.01 or an EB call *P*-value > 2.0, sequencing depth ≥ 30, and variant reads > 4 were adopted. Furthermore, variants only in unidirectional reads; variants in repetitive genomic regions; variants in intergenic, intronic, untranslated, and noncoding RNA regions; and synonymous single-nucleotide variants were excluded. Additionally, known recurrently mutated genes in TFHL, including *RHOA* G17V, *TET2*, *DNMT3A,* and *IDH2,* were manually screened for as additional mutations. Mapping errors were excluded by visual inspection with Integrative Genomics Viewer (IGV) [33]. Detected somatic mutations are listed in Table S5.

Somatic copy number variations (CNVs) were analyzed by the Genome Analysis Tool Kit (GATK; v4.2, Broad Institute, Cambridge, MA, USA) [34]. Buccal samples were used as a panel of normals. The interval list was generated from the target browser extensible data (BED) file of the SureSelect XT Human All Exon V7 kit downloaded from the Agilent website (https://earray.chem.agilent.com/suredesign/index.htm). Gains and losses less than 1 Mb in length were removed from downstream analyses.

Inferring genetic mutations using scRNA-seq data

We applied VarTrix (v1.1.16, 10x Genomics, https://github.com/10XGenomics/vartrix) to extract single-cell variant information from scRNA-seq data. First, BED files of the somatic mutations extracted by the Genomon2 pipeline for WES data were lifted from GRCh37 to GRCh38 with the UCSC web browser (https://genome.ucsc.edu/cgi-bin/hgLiftOver). Then, the BED files converted to variant call format (VCF) were used as input for VarTrix. We ran the

"vartrix" command with --mapq 255, --padding 100, and --umi mode options using a cell barcode file extracted from the integrated Seurat object and a binary alignment map (BAM) file generated by the Cell Ranger pipeline for each sample. Briefly, local sequence alignment at the input variant locus was performed by the Smith-Waterman algorithm, and then wild-type and mutant reads were counted for each cell, respectively. In --umi mode, a consensus is taken across each UMI, and only cases where each UMI exceeded a hardcoded 90% threshold for supporting the same allele were reflected in the results. Owing to the phenomenon commonly occurring in scRNA-seq called "allelic dropout" (insufficient amplification of one allele), it is impossible to judge whether a cell is a "truly wild type (WT)" in the absence of mutant reads [35]. Therefore, we defined a cell as "unknown" if only WT reads or no reads were detected. Cells from THFL patients without WES-identified mutations were classified as "WT" cells.

Estimation of CNVs at the single-cell level

To infer large-scale CNVs from scRNA-seq data, we applied the R package inferCNV (v1.10.1). [36] Non-malignant T cells from each sample were used as controls for analysis of PB and LN tumor cells. We used the following parameters: default de-noise and hidden Markov model (HMM) setting, cutoff = 0.1, cluster_by_groups = FALSE, analysis_mode = 'subclusters', and tumor_subcluster_partition_method = 'random_trees'. Specifically, after removeing noise with the default settings, the random trees method was used to divide cells into groups with consistent CNV patterns, and CNV prediction by HMM was performed at the subcluster level. Uphyloplot2 (v2.3) [37] was applied to visualize phylogenetic trees using the inferCNV output of each sample. CNV scores for each cell were calculated as previously described [38].

Immunohistochemical (IHC) staining

Regarding *PLS3*, which was identified as a candidate tumor cell-specific gene, IHC staining using FFPE samples of tumor tissues was performed by either the Tsukuba Human Tissue Biobank Center Tsukuba Pathological Analysis Support Service (T-PASS, Tsukuba, Japan) or expert hematopathologists at Tokai University School of Medicine. After antigen retrieval with Target Retrieval Solution pH 9.0 (Dako, Santa Clara, CA, USA) using the automated pretreatment system PT Link (Dako; 97°C, 20 min), anti-PLS3 antibody (Sigma-Aldrich) diluted 1:100 was added, and samples were stained with Autostainer Link 48 (Dako). PLS3 staining was determined to be positive when 10% or more of the cells were positive.

High-dimensional spatial analysis by imaging mass cytometry (IMC)

Carrier-free antibodies were manually conjugated to metal isotopes using the MaxPar 8X antibody labeling kit (Standard BioTools Inc., South San Francisco, CA, USA) following the manufacturer's protocol (Maxpar Antibody Labeling Kit PRD002 Version 11). Manually conjugated or pre-metal-tagged antibodies obtained from Standard BioTools Inc. were used for IMC staining (Table S17). FFPE samples from TFHL tissues were incubated in a dry oven for 15 min at 55°C, deparaffinized, and rehydrated using a graded ethanol series. Antigen retrieval was performed using Target Retrieval Solution pH 9.0 (Dako) in a Decloaking Chamber (Biocare Medical, Pacheco, CA, USA) for 10 min at 95°C. Slides were incubated overnight at 4°C with a cocktail of metal-conjugated primary antibodies after blocking with buffer containing 3% BSA. The following day, slides were washed twice with Dulbecco's PBS and counterstained using Cell-ID Intercalator-Ir (Standard BioTools Inc.) for 30 min at RT to visualize the DNA. After a final wash in doubly distilled H_2O, the slides were air-dried for 20

min. IMC data was acquired using a Hyperion imaging system (Standard BioTools Inc.). Subsequently, five regions of interest (ROIs) of 1 500 × 1 500 µm per 1 FFPE sample were identified on consecutive Hematoxylin and Eosin (H&E)-stained slides and ablated by the laser beam.

We converted raw data, exported as MCD files, to the OME-TIFF format and segmented into single cells using the ImcSegmentationPipeline [39]. Specifically, we applied Ilastik (v1.3.3) [40] for pixel classification and generated pixel probability maps for three labels ("nucleus," "cytoplasm," and "background"). Then, cells were segmented based on pixel probabilities and single-cell or image features, such as channel intensities and the number of neighbors, were measured using the CellProfiler (v4.2.1) [41].

The output data from CellProfiler was imported into R (v4.1.0) as a SpatialExperiment object by the imcRtools package (v1.0.2) [42]. After the inverse hyperbolic sine transformation and normalization at the 99th percentile, batch effect correction and unsupervised clustering were performed using the Batchelor and Seurat packages, like with the scRNA-seq data. When performing clustering, four proteins with poor cell-to-cell variability (CD10, E-cadherin, ICOSL, and CD40LG) and two proteins related to the stromal structures (α-smooth muscle actin [SMA] and collagen type I) were excluded. By annotation with canonical markers, we identified a T_{FH} marker-positive cluster thought to be tumor cells and performed re-clustering with immune and stromal cells other than tumor cells. Next, we computed the averaged cell-cell interaction counts and compared them to a null distribution of counts generated by random permutations to estimate the interaction or avoidance between cell types using the "testInteractions" function of imcRtools (Table S19). Finally, the pixel- and cell-level visualization of each image was performed by the cytomapper package (v1.6.0) [43].

Code Availability

The code used for data analysis are available from the corresponding author upon request (sakatama@md.tsukuba.ac.jp [M.S.-Y.]).

Supplementary Notes

Supplementary Note 1: Related to "Expansion of dysfunctional CD8$^+$ and regulatory T cells in LNs" and "Clonal tracking between T cells in LNs and PB"

The following is a comment regarding subclustering and the TCR analysis of non-malignant PB T cells.

Subclustering of non-malignant PB T cells revealed a similar immunophenotype to those of LNs. Specifically, the proportions of CD8 T_{DYS} and $T_{PRO/DYS}$ were increased, whereas those of CD4/CD8 T_N and T_{CM} were decreased in patients with TFHL compared with those in HDs (Fig. S10A–B). Although the increase in the proportion of T_{REG} was not significant, the proportion of proliferating T_{REG} and the expression levels of T_{REG} activation markers such as *FOXP3*, *BATF*, *TNFRSF4,* and *TNFRSF18* [44] and co-inhibitory molecules such as *PDCD1* and *TIGIT* were higher in the T_{REG} of patients with TFHL than those of HDs (Fig. S10C–F).

By TCR repertoire analysis of non-malignant T cells in PB, we found that effector CD8$^+$ T cells (effector memory [T_{EM}] and effector memory cells re-expressing CD45RA [T_{ERMA}]) were clonally expanded in both patients with TFHL and HDs, whereas CD8 T_{DYS}/$T_{PRO/DYS}$ were clonally expanded only in the patients with TFHL (Fig. S17A). TCR overlap analysis showed that CD8 T_{DYS} and CD8 $T_{PRO/DYS}$ shared their TCRs in TFHL PB while no shared TCRs were detected between effector and dysfunctional CD8$^+$ T cells of TFL PB, unlike the LN data (Fig. S17B). In addition, over 50% of known, pathogen-specific CDR3s, such as cytomegalovirus

(CMV), Epstein-Barr virus (EBV), and influenza virus detected by collation with VDJdb database [45], were concentrated in effector $CD8^{+}$ T cells in both TFHL patients and HDs, suggesting that they were “bystander” $CD8^{+}$ T cells that recognized antigens unrelated to tumor cells (data not shown).

Supplementary Note 2: Related to “*In-silico* and spatial intercellular interactions and formation of the TFHL immune-evasive microenvironment”

The following is a comment regarding the subclustering and annotation of non-malignant cells in the spatial analysis.

By unsupervised clustering and annotation using canonical markers, we identified 14 subclusters: granzyme B $(GZMB)^{low}$ $PD1^{high}$ CD8 T_{DYS}, $GZMB^{high}$ $PD1^{low}$ CD8 T_{EFF}, $FOXP3/CD25^{+}$ T_{REG}, $CD3/CD4^{+}$ or $CD3/CD8^{+}$ T cells, $CD19^{+}$ B cells, $CD3^{-}$ $CD4^{dim}$ $CD14^{+}$ myeloid cells, $CD3/CD4/CD19^{-}$ $CD132/CD11B^{+}$ NK cells, and $CD21/CD35^{+}$ follicular dendritic cells (FDCs) (Table S18). Cluster 10 showed $CD20^{-}$ $CD19/CD79A^{dim}$, annotated as PBL/PC (Fig. S18A). Cluster 13 exhibited high expressions of α-SMA and collagen type I, whereas lineage markers were negative (Fig. S18B), suggestive of stromal cells such as endothelial cells of blood and lymphatic vessels.

Consistent with previous reports on AITL [46], FDCs formed meshwork structures and were identified as indistinguishable clusters from surrounding cells (clusters 3, 6, and 11; Fig. S18C). Moreover, B cells adjacent to tumor cells were recognized as a single cluster with tumor cells (cluster 12; Fig. S18D).

Supplementary Table Legends

Supplementary Table 1. Characteristics of patients with TFHL and controls and comparison of patient cohorts.

Supplementary Table 2. IMC analysis of TFHL tumor tissues.

Supplementary Table 3. Summary of tumor-cell clonotypes detected by scTCR-seq.

Supplementary Table 4. Proportion of cells detected by scRNA-seq and FCM analysis.

Supplementary Table 5. Somatic mutations detected by WES of 14 patients with TFHL.

Supplementary Table 6. Marker genes for each subcluster of LN and PB tumor cells.

Supplementary Table 7. Somatic mutations detected by reanalysis of scRNA-seq.

Supplementary Table 8. DEGs upregulated in LN and PB tumor cells compared with all MNCs and normal T_{FH} cells.

Supplementary Table 9. DEG analysis of three candidate genes for novel tumor cell-specific markers compared with all non-malignant MNCs.

Supplementary Table 10. IHC staining of PLS3 on PTCLs and B-cell lymphomas.

Supplementary Table 11. DEGs between T_{REG} of RR TFHL LNs and HLNs.

Supplementary Table 12. DEGs between *FCRL4*$^{+}$ MBCs (B2) and *FCRL4*$^{-}$ MBCs (B1) of LNs.

Supplementary Table 13. DEGs in *FCRL4*$^{+}$ MBCs (B2) of ND or RR TFHL LNs compared with those of HLNs.

Supplementary Table 14. AITL B-specific gene set cited from Fujisawa et al. (2022) [1] for GSEA.

Supplementary Table 15. DEGs in *XCL1*$^{+}$ NK cells (NK1) of ND TFHL LNs compared with those of HLNs.

Supplementary Table 16. DEGs in *XCL1*$^{+}$ NK cells (NK1) of RR TFHL LNs compared with those of HLNs.

Supplementary Table 17. Antibodies used for IMC analysis.

Supplementary Table 18. Markers and annotation of each cluster for subclustering of non-malignant cells in IMC analysis.

Supplementary Table 19. Results of spatial interaction analysis.

Supplementary Table 20. Antibodies used for FCM analysis and IHC staining.

Supplementary Figure 1

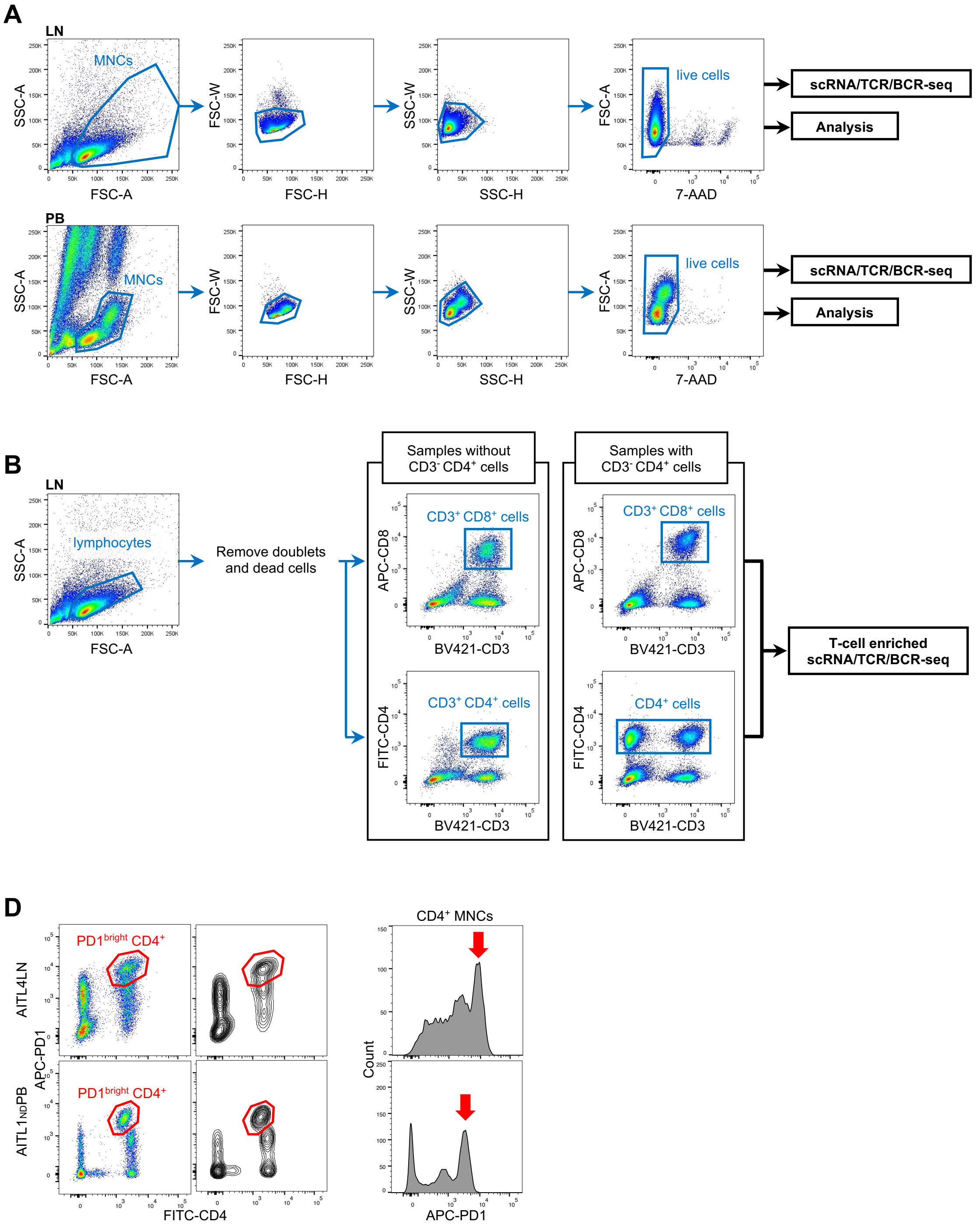

Figure S1. Cell-surface protein expression analysis and sorting by flow cytometry (FCM)

(A) Mononuclear cell (MNC) sorting of lymph node (LN) and peripheral blood (PB) samples. After removing doublets and dead cells by forward versus side scatter plot and 7-Amino-Actinomycin D (7-AAD) staining, MNCs were sorted, and library construction of single-cell RNA and T-/B-cell receptor (TCR/BCR) sequencing (scRNA/TCR/BCR-seq) or FCM analysis were performed. FSC, forward scatter; SSC, side scatter.

(B) CD4/$CD8^+$ T-cell sorting for T cell-enriched library. For samples in which tumor-containing $CD3^-$ $CD4^+$ cells were detected in the lymphocyte fraction in the initial analysis, $CD3^+$ $CD8^+$ cells and $CD4^+$ cells were sorted.

(C) Cytokeratin staining for homeostatic LNs (HLNs). Cytokeratin negativity confirmed the absence of tumor-cell contamination.

(D) Detection of tumor cells by surface antigen. Tumor cells were detected as a $PD1^{bright}$ $CD4^+$ T-cell population, as previously reported [47].

Supplementary Figure 2

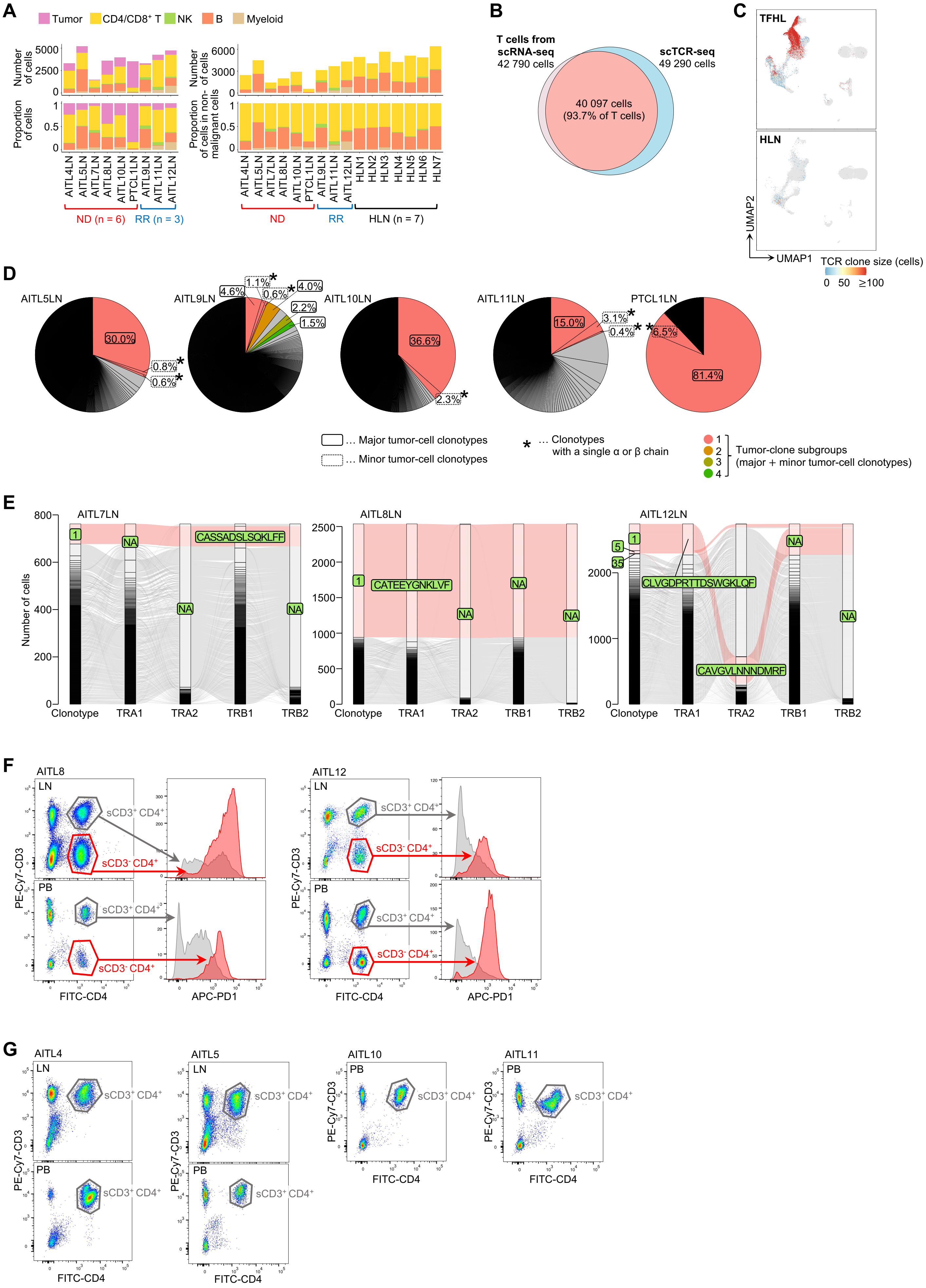

Figure S2. Subclustering and TCR repertoire analysis of LN samples

(A) Absolute number (top of panels) and proportion (bottom of panels) of cells within all LN MNCs (left panel) and non-malignant LN MNCs (right panel) for each sample, color-coded by cell types. B, B cell; CD4/CD8 T, $CD4^+$ and $CD8^+$ T cell; Myeloid, myeloid cell; NK, natural killer cell; ND, newly diagnosed T follicular helper cell lymphoma (TFHL); RR, relapsed or refractory TFHL; Tumor, tumor cell.

(B) Comparison between T cells from scRNA-seq data and TCRs from scTCR-seq data of LN samples.

(C) Clone size of TCRs in LNs. The number of cells expressing each clonotype was defined as clone size and illustrated for each cell.

(D) Pie charts of TCR clonotypes for each sample. Tumor clones are colored.

(E) Sankey plots of each clonotype in cases where the major tumor-cell clonotype was a TCR with a single chain. The major clonotypes are colored in red and green, showing the CDR3 sequences per TCR chain. "NA" indicates that no TCR chain was detected. NA, not available; TRA, TCR alpha chain; TRB, TCR beta chain.

(F) Relationship between $CD3^-$ $CD4^+$ cells and the expression level of PD1 in each sample. $CD3^-$ $CD4^+$ (red) and $CD3^+$ $CD4^+$ (grey) populations detected by a CD3 versus CD4 plot (left panel) are plotted separately on a histogram of PD1 expression (right panel). Samples in which $CD3^-$ $CD4^+$ cells were detected by FCM analysis of LN samples are shown. sCD3ε, surface CD3ε.

(G) FCM plots of CD3 versus CD4 for TFHL patients without single-chain TCR expansion. Since LN samples from AITL10 and AITL11 were not available for detailed FCM analysis, only PB FCM data are shown for these patients.

Supplementary Figure 3

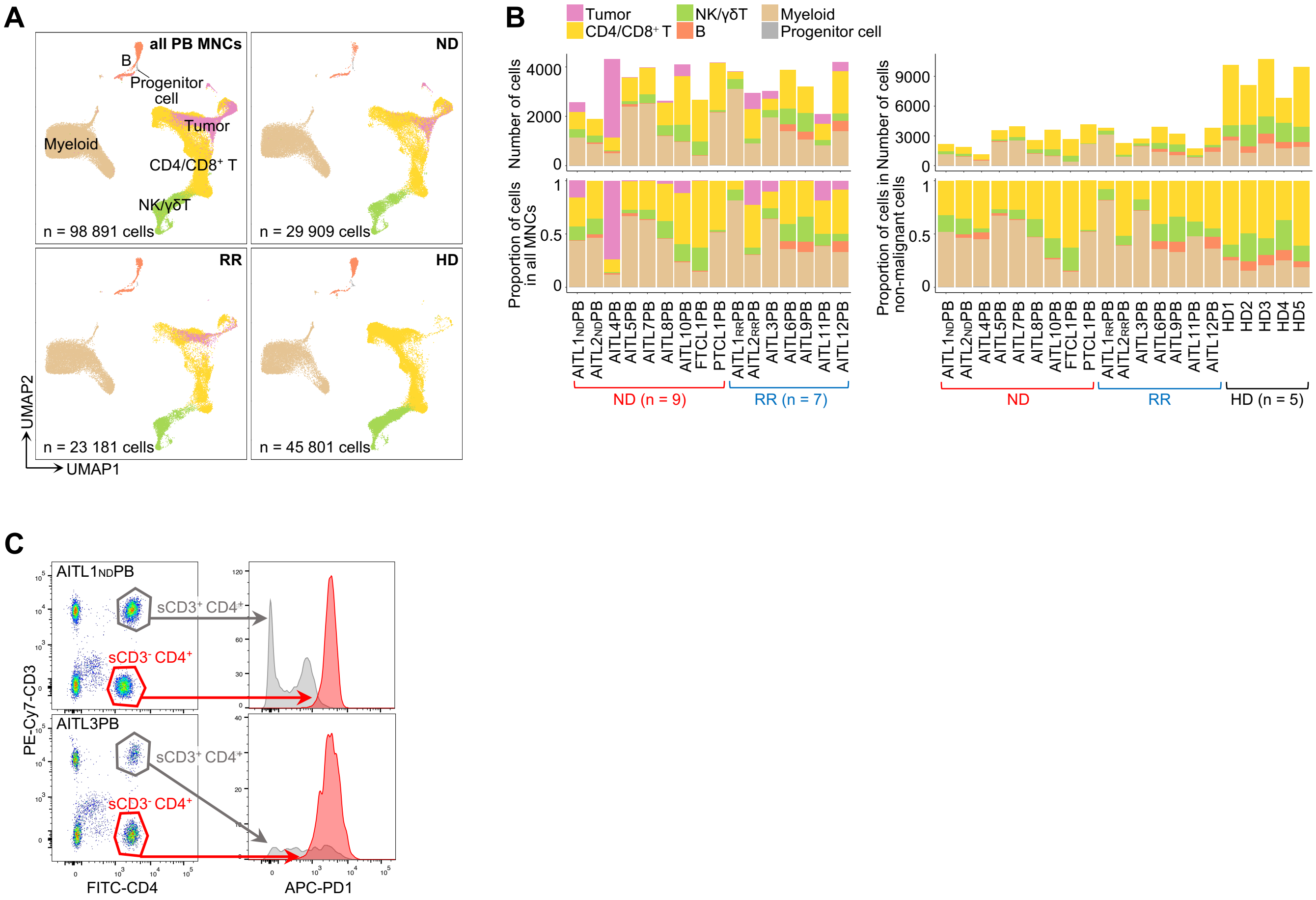

Figure S3. Single-cell analysis of PB samples

(A) Uniform Manifold Approximation and Projection for Dimension Reduction (UMAP) plots of all PB MNCs from 16 TFHL and 5 healthy donor (HD) PB samples, colored according to cell type (top left). Cells are shown separately for each clinical status (top right and bottom). NK/γδT, natural-killer and gamma-delta T cell.

(B) Absolute number (top of panels) and proportion (bottom of panels) of cells within all PB MNCs (left panel) and non-malignant PB MNCs (right panel) for each sample, color-coded by cell types.

(C) Relationship between $CD3^{-}$ $CD4^{+}$ cells and the expression level of PD1 in PB samples. $CD3^{-}$ $CD4^{+}$ (red) and $CD3^{+}$ $CD4^{+}$ (grey) populations detected by a CD3 versus CD4 plot (left panel) are plotted separately on a histogram of PD1 expression (right panel). Samples in which $CD3^{-}$ $CD4^{+}$ cells were detected by FCM analysis of PB samples are shown.

Supplementary Figure 4

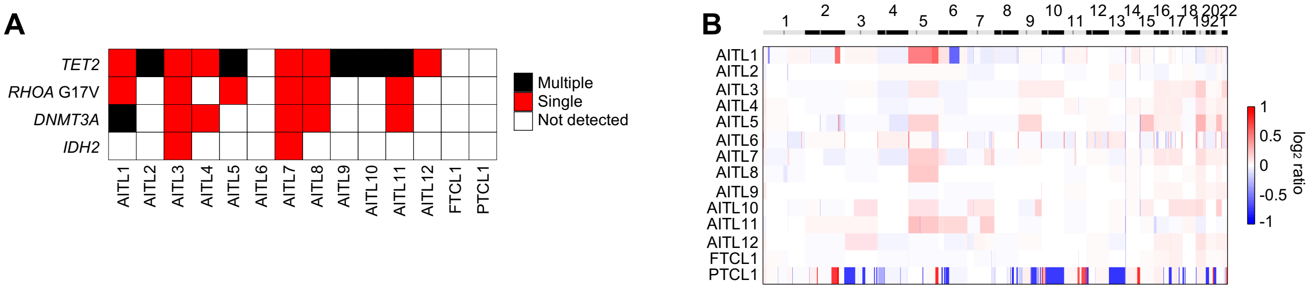

Figure S4. Bulk genomic profiling of the 14 TFHL patients

(A) Recurrent gene mutations detected by whole-exome sequencing (WES). Red squares indicate single mutations and black ones indicate multiple mutations.

(B) Copy number variation (CNV) analysis using WES data from tumor tissue samples. Significant CNVs were called by the Genome Analysis Tool Kit [34].

Supplementary Figure 5

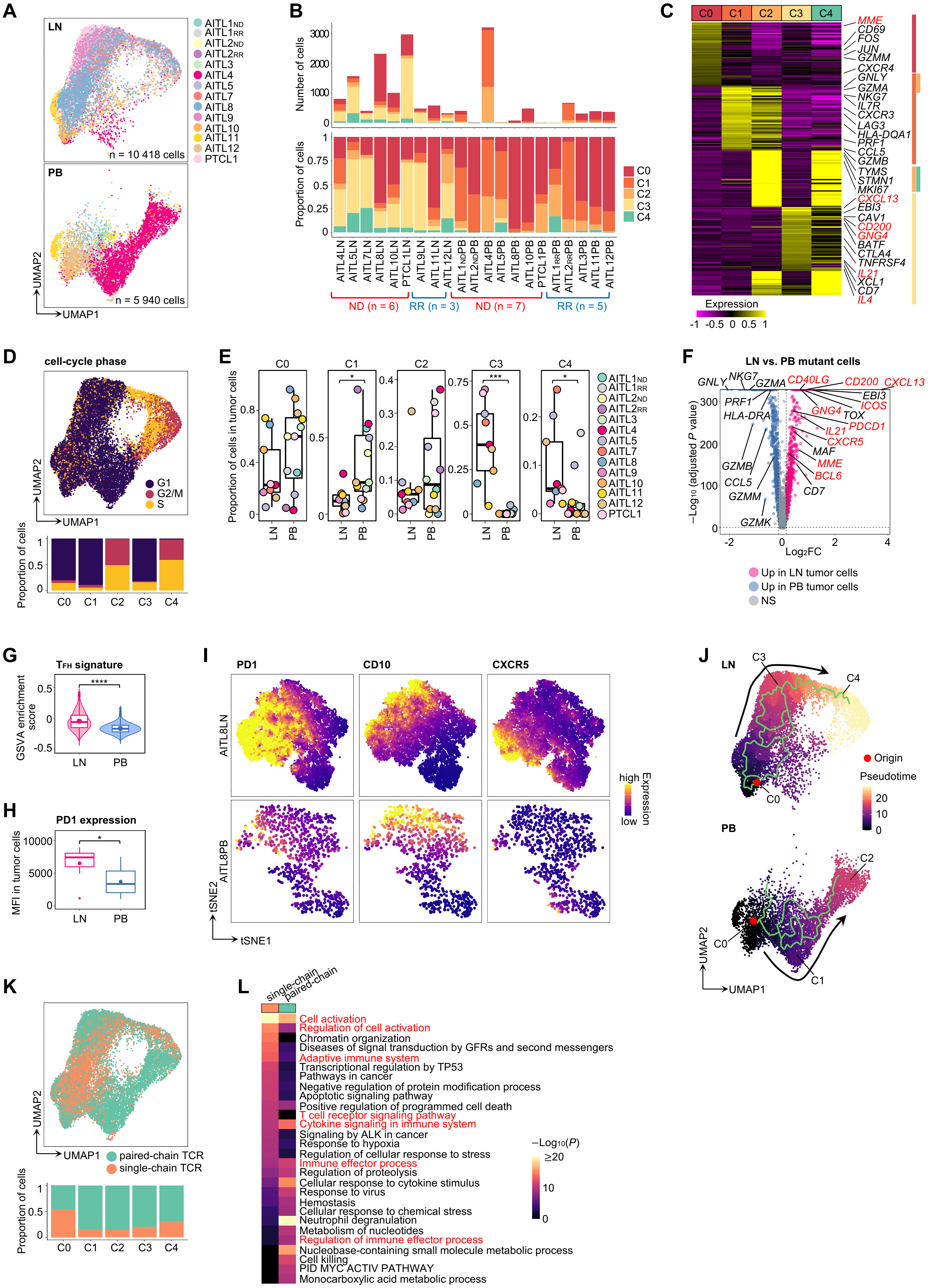

Figure S5. Subclustering of tumor cells from LN and PB samples

(A) UMAP plots of LN and PB tumor cell subclusters. Cells are color-coded by each sample and shown separately for each tissue.

(B) Absolute number (top) and proportion within all tumor cells (bottom) of cells for each sample, color-coded by tumor subclusters.

(C) Heatmap of the top 50 differentially expressed genes (DEGs) for each subcluster. T follicular helper (T_{FH}) markers are shown in red.

(D) Feature plot showing estimation of cell cycle phase. Cell cycle score calculations were performed based on canonical markers using the "CellCycleScoring" function of Seurat, and each cell is colored according to the estimated cell cycle phase.

(E) Comparison of the proportion of each cluster in LN and PB tumor cells. The boxplots show the median (center line), interquartile range (box limits), minimum to max values (whiskers), and samples (dots) for each group. *P*-values are shown only when there is a significant difference.

(F) Volcano plot of DEGs between LN (pink) and PB (blue) tumor cells. NS, not significant; vs., versus.

(G) Comparison of gene set variation analysis (GSVA) enrichment score for T_{FH} signature in LN (pink) and PB (blue) tumor cells. The boxplots show the median (center line), mean (center dot), interquartile range (box limits), and minimum to max values (whiskers) for each group.

(H) Comparison of PD1 expression levels by FCM in LN (pink) and PB (blue) tumor cells. Tumor cells were detected as a $PD1^{bright}$ $CD4^{+}$ T cell population by FCM [47]. The boxplots show the median (center line), mean (center dot), interquartile range (box limits), and minimum to max values (whiskers) for each group. MFI, median fluorescence intensity.

(I) t-Distributed Stochastic Neighbor Embedding (t-SNE) plots of FCM analysis for tumor cells of AITL8LN (top) and AITL8PB (bottom), colored by the expression levels of T_{FH} markers

(PD1, CD10, and CXCR5).

(J) Trajectory analysis by Monocle3 [27] for LN (top) and PB (bottom) tumor cells. The red points were set as the origins of trajectory.

(K) UMAP plot showing tumor cells with paired-chain (green) and single-chain (orange) TCRs (top) and their proportions in each cluster (bottom).

(L) Heatmap of gene ontology (GO) analysis of tumor cells with paired-chain (green) and single-chain (orange) TCRs.

$^{*}P < 5.0 \times 10^{-2}$, $^{***}P < 1.0 \times 10^{-3}$, $^{****}P < 1.0 \times 10^{-4}$.

Supplementary Figure 6

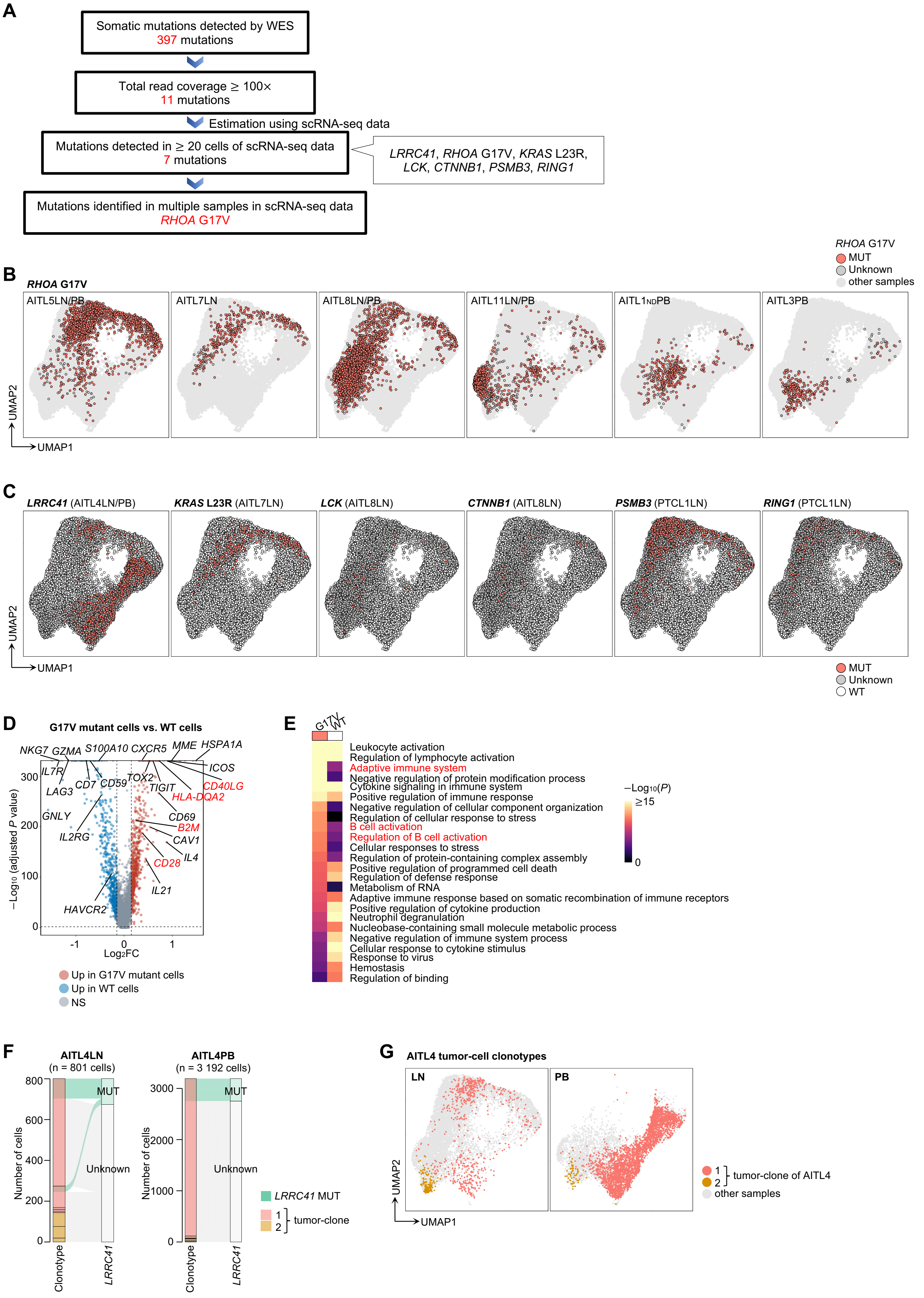

Figure S6. Estimation of genetic mutations using single-cell data

(A) Overview of reanalysis of somatic mutations using scRNA-seq data.

(B) Distribution of *RHOA* G17V (G17V) for each sample in LN and PB tumor cells. MUT, mutant cells; Unknown, cells with no mutant reads or no coverage.

(C) Distribution of detectable mutations other than G17V (*LRRC41*, *KRAS* L23R, *LCK*, *CTNNB1*, *PSMB3,* and *RING*) in LN and PB tumor cells. The sample names in parentheses show the samples in which the mutations were detected. WT, wild-type cells.

(D) Volcano plot of DEGs between G17V mutant (red) and WT (blue) cells in LN and PB tumor cells. G17V unknown cells were removed from the analysis.

(E) Heatmap of GO analysis of G17V mutant (red) and WT (white) tumor cells.

(F) Relationship between *LRRC41* mutant cells and their clonotypes in AITL4LN/PB tumor cells. In the Sankey plot, tumor clones of AITL4 and *LRRC41* mutant cells (green) are colored.

(G) UMAP plot showing the distribution of AITL4 tumor clones.

Supplementary Figure 7

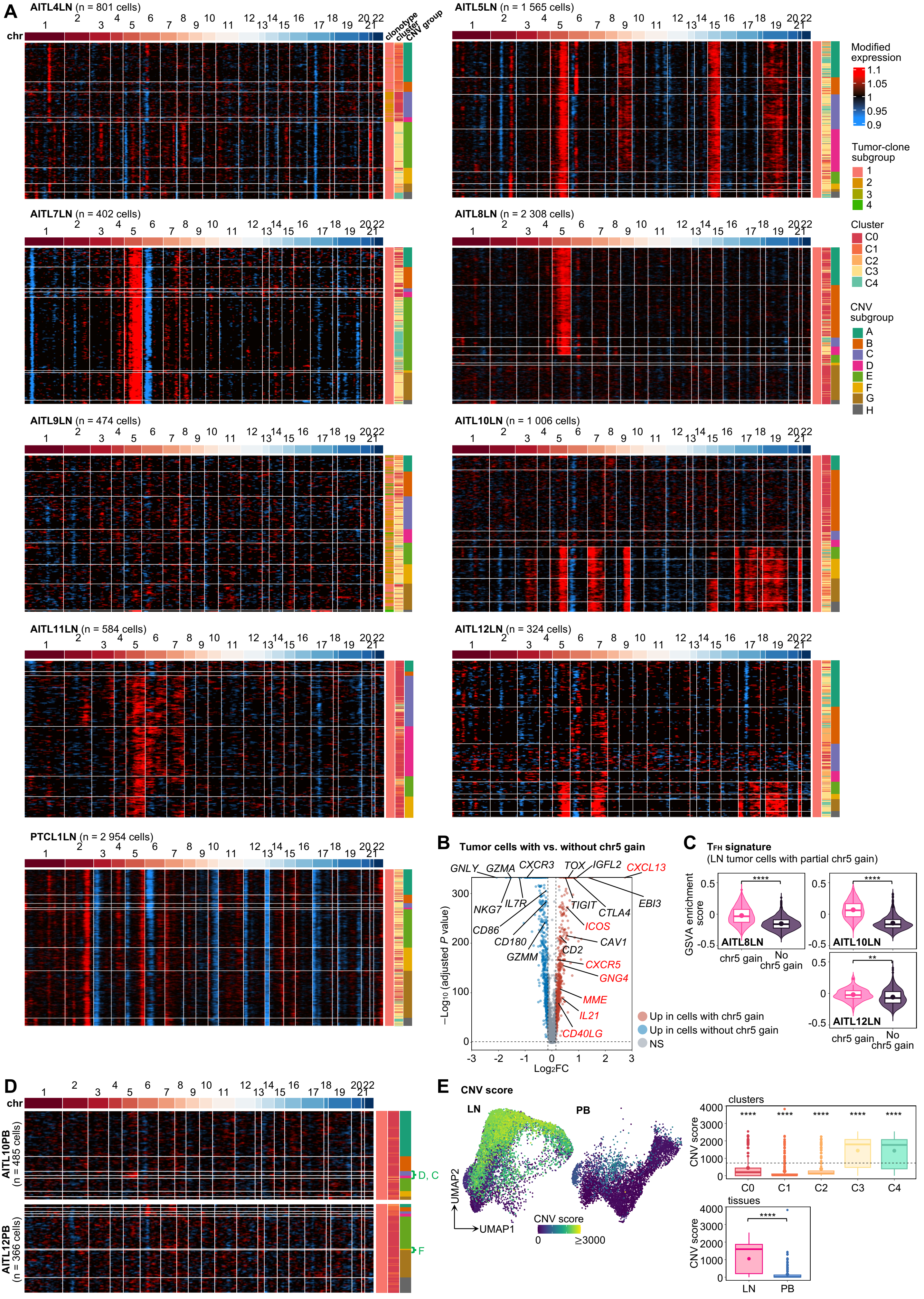

Figure S7. Inferring CNVs using single-cell data

(A) Heatmaps of estimated CNVs for LN tumor cells by inferCNV [36]. Right bars represent tumor clones, subclusters of tumor cells, and subgroups based on CNV patterns, respectively. chr, chromosome.

(B) Volcano plot of DEGs between tumor cells with chr5 gain (red) and those without (blue).

(C) Comparison of GSVA enrichment score for the T_{FH} signature between tumor cells with chr5 gain (pink) and those without (blue) in TFHL LNs with partial chr5 gain.

(D) Heatmaps of estimated CNVs for PB tumor cells in AITL10 and AITL12.

(E) Distribution (left) and comparison for each cluster (top right) or tissue (bottom right) of CNV scores estimated using scRNA-seq data. In the boxplots of the top right panel, the dotted line represents the mean CNV scores across all clusters and adjusted *P*-values are calculated by the pairwise Wilcoxon test for each cluster against the mean value of all clusters.

All boxplots show the median (center line), mean (center dot), interquartile range (box limits), and minimum to max values (whiskers) for each group. $^{**}P < 1.0 \times 10^{-2}$, $^{****}P < 1.0 \times 10^{-4}$.

Supplementary Figure 8

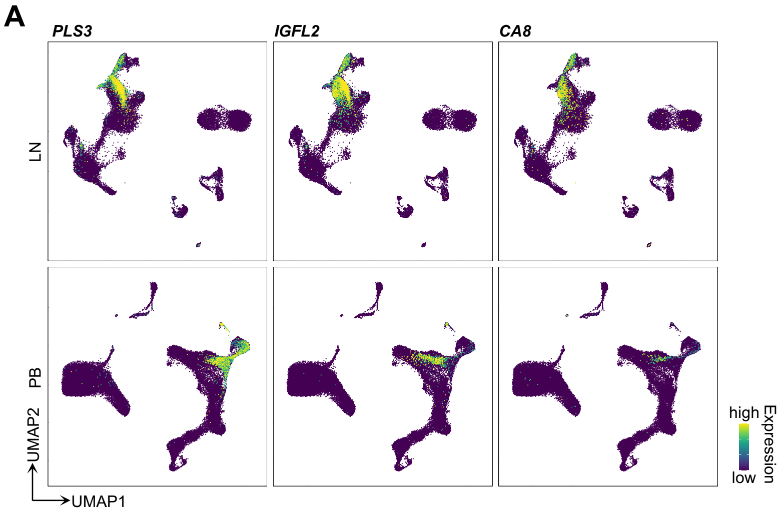

Figure S8. Identification of novel tumor-specific cell markers

(A) Feature plots of candidate marker genes in all LN (top) and PB (bottom) MNCs.

Supplementary Figure 9

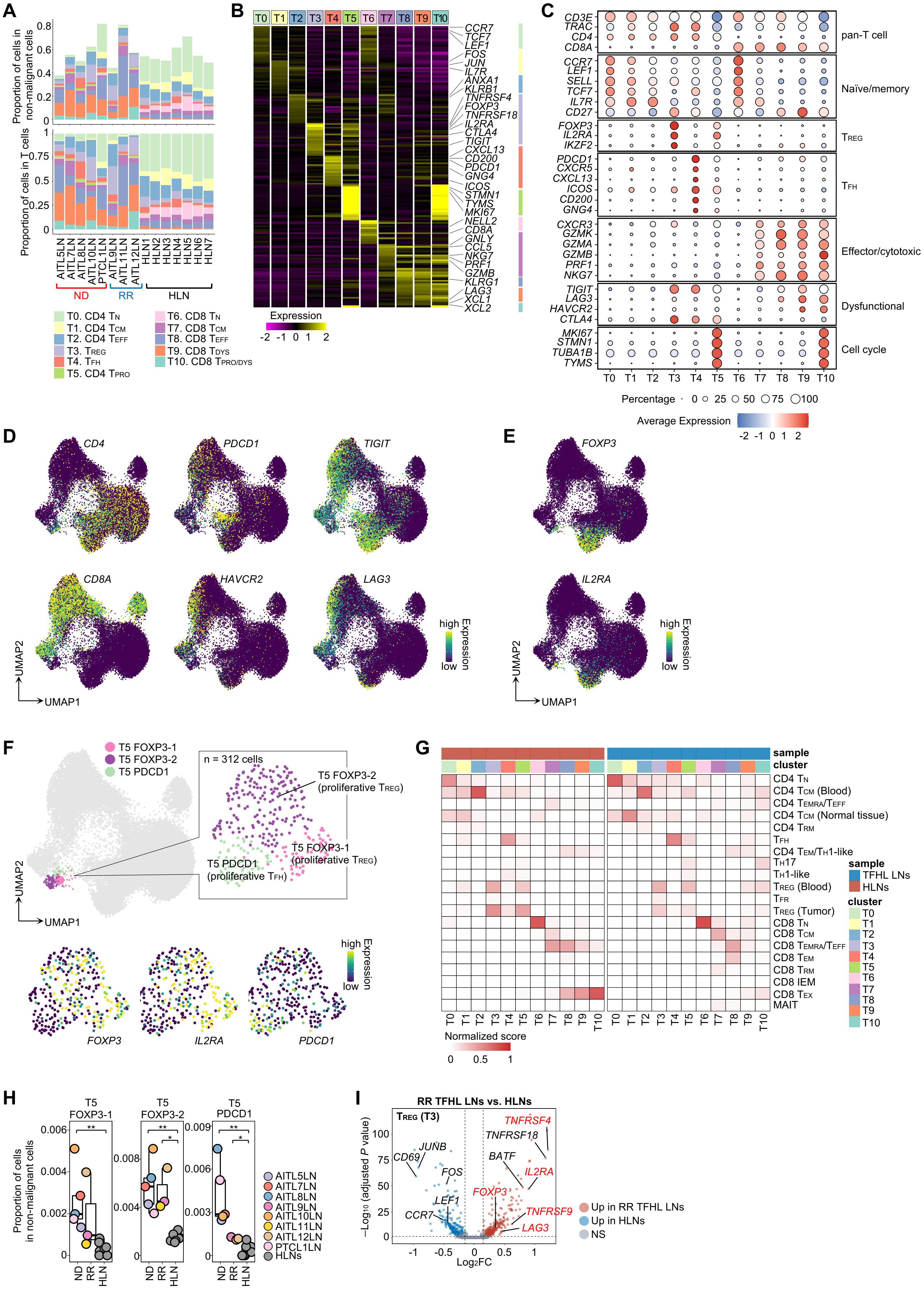

Figure S9. Annotation of subclusters of non-malignant LN T cells

(A) Proportion of each cluster within non-malignant MNCs (top) and within non-malignant T cells (bottom) for each sample. T_{CM}, central memory T cell; T_{DYS}, dysfunctional T cell; T_{EFF}, effector T cell; T_N, naïve T cell; T_{PRO}, proliferative T cell; $T_{PRO/DYS}$, proliferative dysfunctional T cell; T_{REG}, regulatory T cell.

(B) Heatmap of the top 20 DEGs for each cluster of non-malignant LN T cells.

(C) Average expression per cluster of selected markers for cell-type annotation in non-malignant LN T cells.

(D) Feature plots of dysfunctional T-cell markers.

(E) Feature plots of T_{REG} markers.

(F) Subclustering of the CD4 T_{PRO} (T5) cluster. UMAP plot (top) and feature plots of T_{REG} and T_{FH} markers (bottom).

(G) Cell-type estimation scores by SingleR [13] using previously published single-cell data as a reference [48] for each subcluster of non-malignant T cells.

(H) Comparison of proportions of each subcluster of CD4 T_{PRO} in non-malignant MNCs of each sample. The boxplots show the median (center line), interquartile range (box limits), minimum to max values (whiskers), and samples (dots) for each group. *P*-values are shown only for significant differences. $*P < 5.0 \times 10^{-2}$, $**P < 1.0 \times 10^{-2}$.

(I) Volcano plot of DEGs in T_{REG} from RR TFHL LNs (red) and those from HLNs (blue).

Supplementary Figure 10

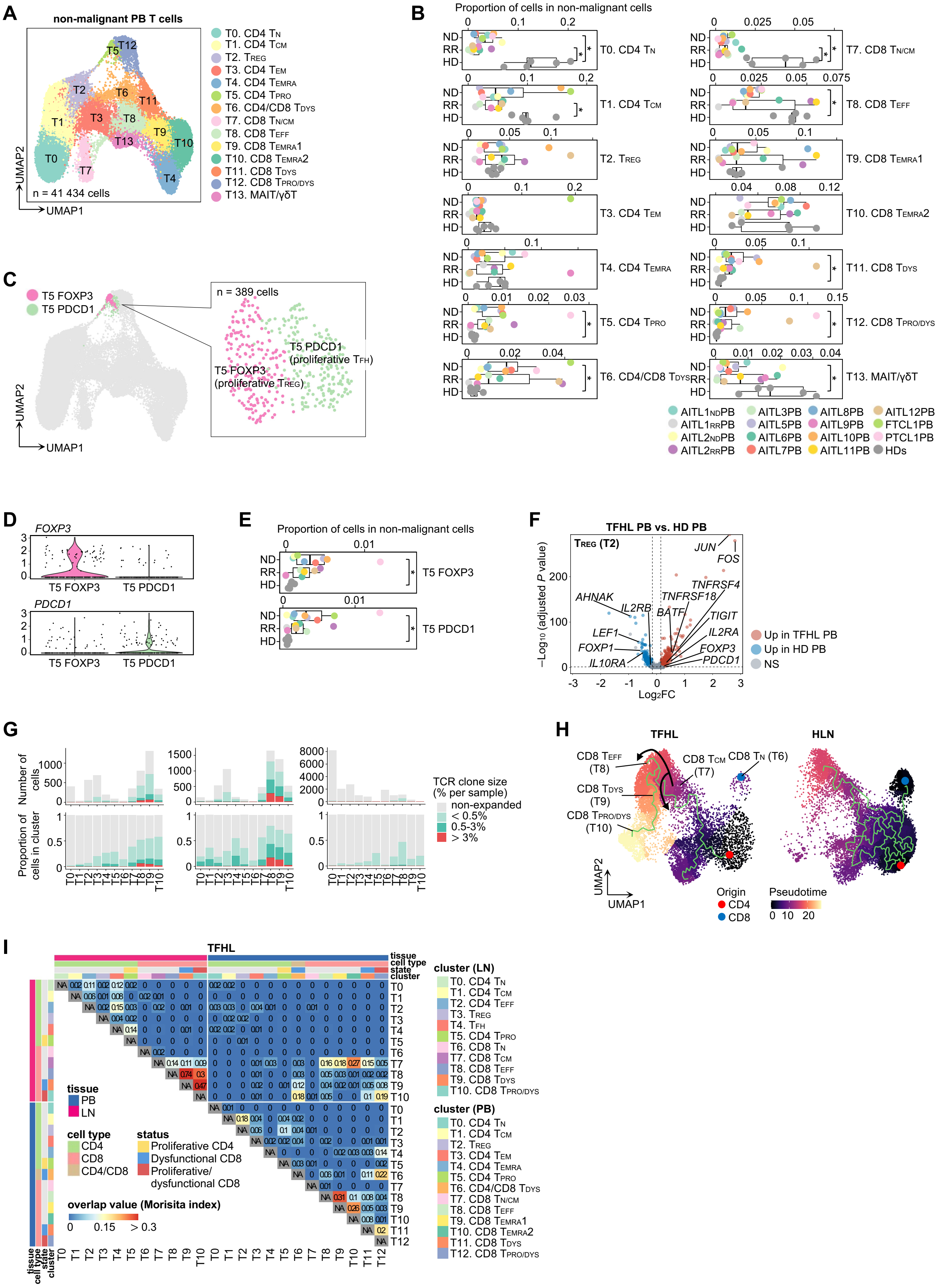

Figure S10. Subclustering of non-malignant PB T cells and non-malignant TCR repertoire analysis

(A) UMAP plot of non-malignant PB T-cell subclusters. MAIT/γδT, mixture of mucosal-associated invariant T cells and gamma-delta T cells; T_{EM}, effector memory T cell; T_{EMRA}, effector memory T cells re-expressing CD45RA; $T_{N/CM}$, mixture of naïve T cells and memory T cells.

(B) Comparison of proportions of each cluster in non-malignant MNCs of each sample. *P*-values are shown only for significant differences.

(C,D) Subclustering of CD4 T_{PRO} (T5) cluster from non-malignant PB T cells. UMAP plot (C) and expression levels of *FOXP3* and *PDCD1* (D).

(E) Comparison of proportions of each T5 subcluster in non-malignant MNCs of each sample. *P*-values are shown only for significant differences.

(F) Volcano plot of DEGs between PB T_{REG} of TFHL patients and those of HDs.

(G) Clone size of TCRs in non-malignant LN T cells. The proportion of each clonotype in all TCRs of non-malignant T cells was calculated for each sample and shown separately for each cluster. Non-expanded, clonotypes were expressed in < 2 cells.

(H) Trajectory analysis by Monocle3 [27] for non-malignant LN T cells. The red and blue points were set as the origin of $CD4^+$ and $CD8^+$ T cells, respectively.

(I) TCR overlap analysis between LN (pink) and PB (blue) non-malignant T cells of TFHL patients.

All boxplots show the median (center line), interquartile range (box limits), minimum to max values (whiskers), and samples (dots) for each group. $^*P < 5.0 \times 10^{-2}$.

Supplementary Figure 11

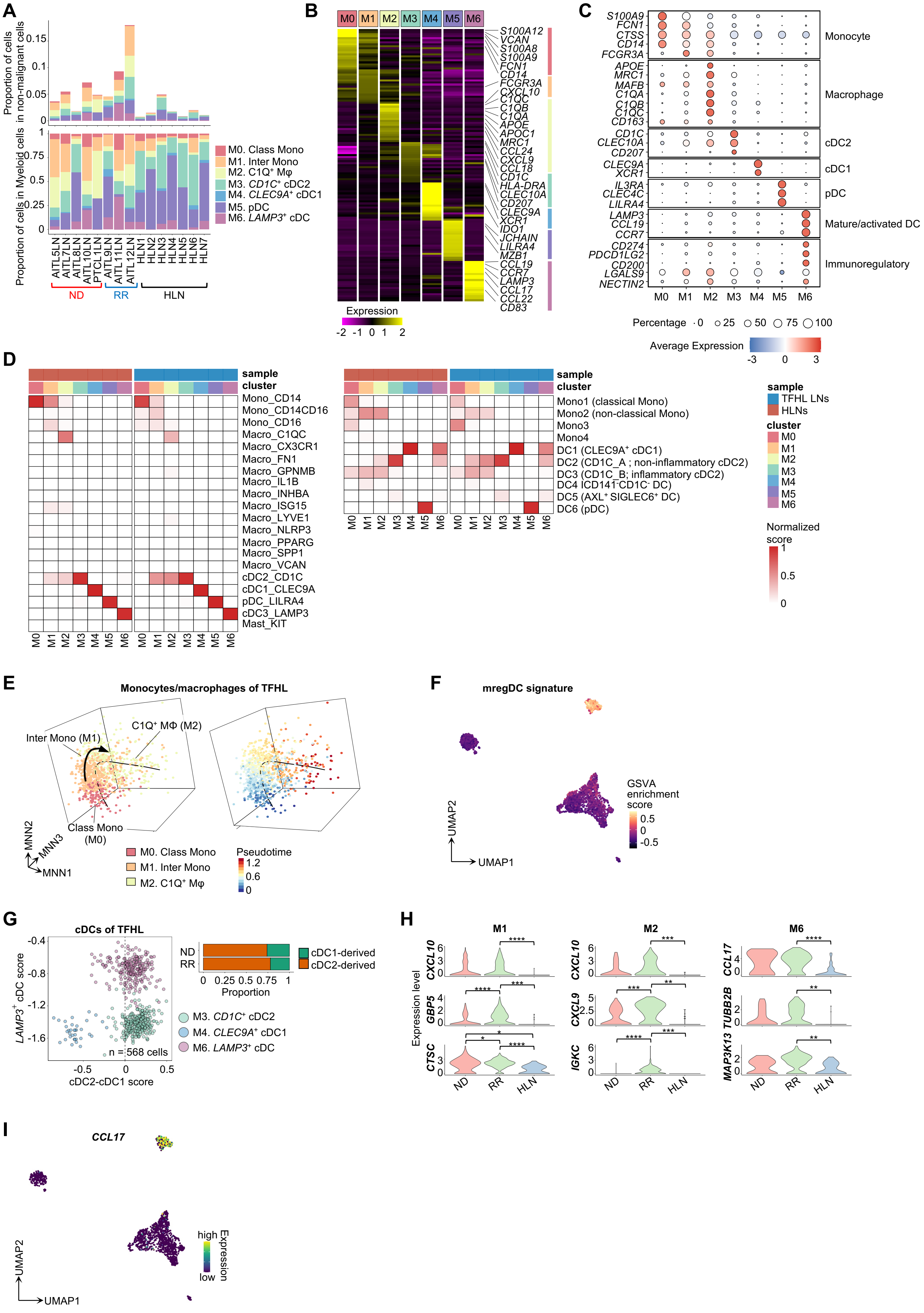

Figure S11. Annotation of LN myeloid-cell subclusters

(A) Proportions of each LN myeloid-cell subcluster within non-malignant MNCs (top) and myeloid cells (bottom) for each sample. $C1Q^{+}$ Mφ, complement component C1q positive macrophage; $CD1C^{+}$ cDC2, CD1C-positive type2 conventional dendritic cell; Class Mono, classical monocyte; *CLEC9A*$^{+}$ cDC1, *CLEC9A*-positive type1 cDC; Inter Mono, intermediate monocyte; *LAMP3*$^{+}$ cDC, *LAMP3*-positive cDC; pDC, plasmacytoid DC.

(B) Heatmap of the top 20 DEGs for each cluster of LN myeloid cells.

(C) Average expression per cluster of selected markers for cell-type annotation in LN myeloid cells.

(D) Cell-type estimation scores by SingleR [13] for each cluster of LN myeloid cells using scRNA-seq data from Cheng et al. [9] (left) and Villani et al. [5] (right).

(E) Trajectory inference by Slingshot [28] for LN monocytes and macrophages (M0–2) of TFHL, color-coded by cluster (left) and pseudo-time (right).

(F) GSVA enrichment scores for “mature DC enriched in immunoregulatory molecules (mregDC)” signature [49] in LN myeloid cells.

(G) Estimation of the origin of *LAMP3*$^{+}$ cDCs of TFHL using a previously reported scoring system [9,49].

(H) Violin plots of the top 3 DEGs upregulated in myeloid subclusters increasing in RR TFHL LNs compared with those of HLNs. *P*-values are shown only for significant differences. $*P < 5.0 \times 10^{-2}$, $**P < 1.0 \times 10^{-2}$, $***P < 1.0 \times 10^{-3}$, $****P < 1.0 \times 10^{-4}$.

(I) Feature plot of *CCL17* in LN myeloid cells from TFHL patients.

Supplementary Figure 12

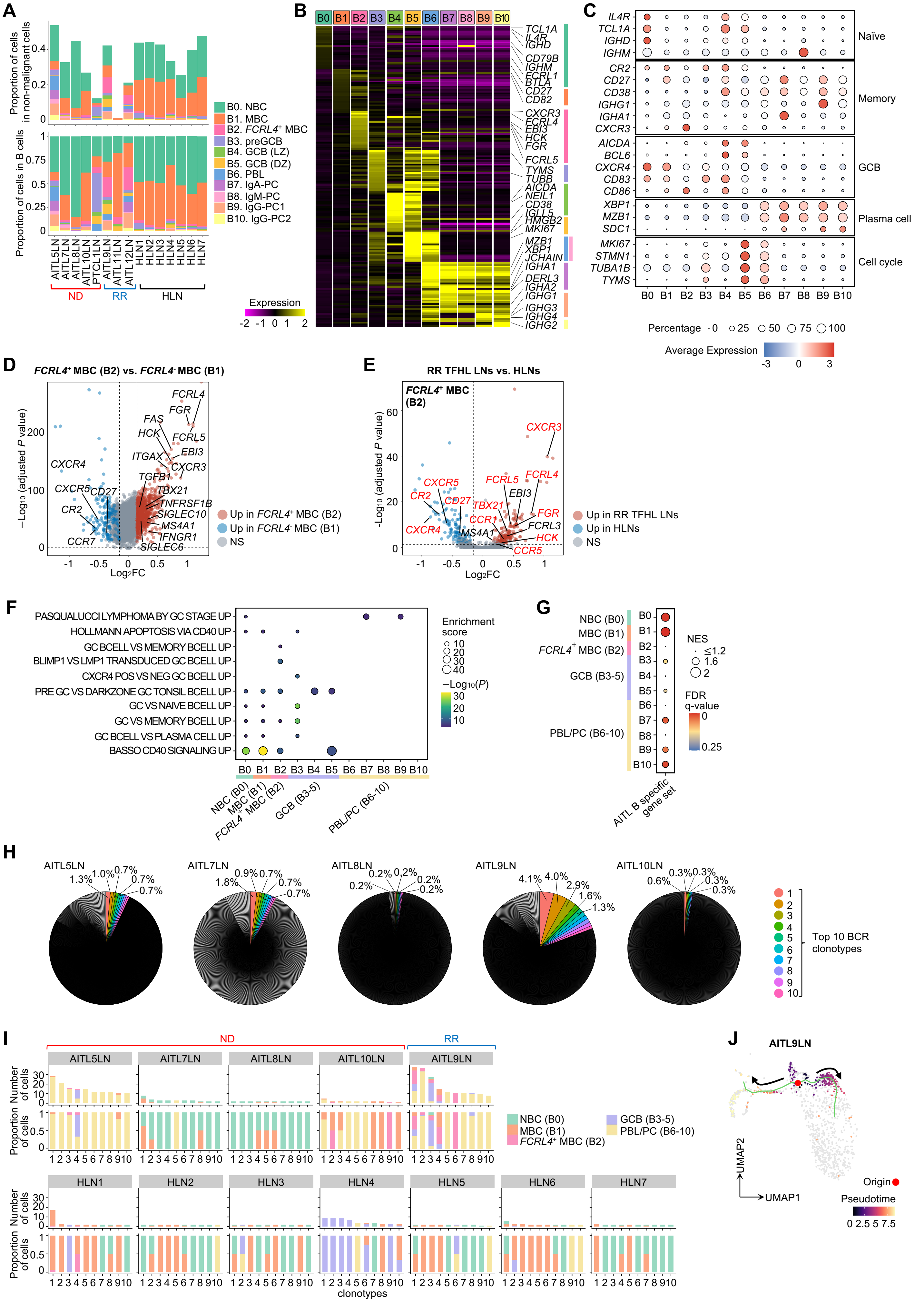

Figure S12. Annotation of LN B-cell subclusters

(A) Proportions of each LN B-cell subcluster within non-malignant MNCs (top) and within B cells (bottom) for each sample. *FCRL4*$^+$ MBC, *FCRL4*-positive memory B cell; GCB (DZ), germinal center B cell in the dark zone; GCB (LZ), GCB in the light zone; MBC, memory B cell; NBC, naïve B cell; PBL, plasmablast; PC, plasma cell; preGCB, pre-germinal center B cell.

(B) Heatmap of the top 25 DEGs for each cluster of LN B cells.

(C) Average expression per cluster of selected markers for cell-type annotation in LN B cells.

(D) Volcano plot of DEGs in *FCRL4*$^+$ MBCs (B2, red) and *FCRL4*$^-$ MBCs (B1, blue) of LNs.

(E) Volcano plot of DEGs in *FCRL4*$^+$ MBCs from RR TFHL LNs (red) and those from HLNs (blue).

(F) Dot plot of GO analysis of DEGs upregulated in B cells from TFHL LNs compared with those from HLNs for GCB-related pathways analyzed by Fujisawa et al. [1].

(G) Dot plot of gene set enrichment analysis for an AITL B-specific gene set previously reported [1]. Cut-off, false discovery rate (FDR) q-value < 0.25; NSE, normalized enrichment score.

(H) Pie charts of BCR clonotypes for each TFHL sample. The top 10 BCRs are colored and the proportions of the top 5 BCRs are shown.

(I) Clone sizes of the top 10 BCR clonotypes for each LN sample. The number (top) and proportions (bottom) of cells expressing each clonotype were shown color-coded by cell types.

(J) Trajectory analysis by Monocle3 [27] for the cells sharing BCRs with *FCRL4*$^+$ MBCs in AITL9LN. The red point was set as the origin of the trajectory. The cells without sharing the BCRs are shown in gray.

Supplementary Figure 13

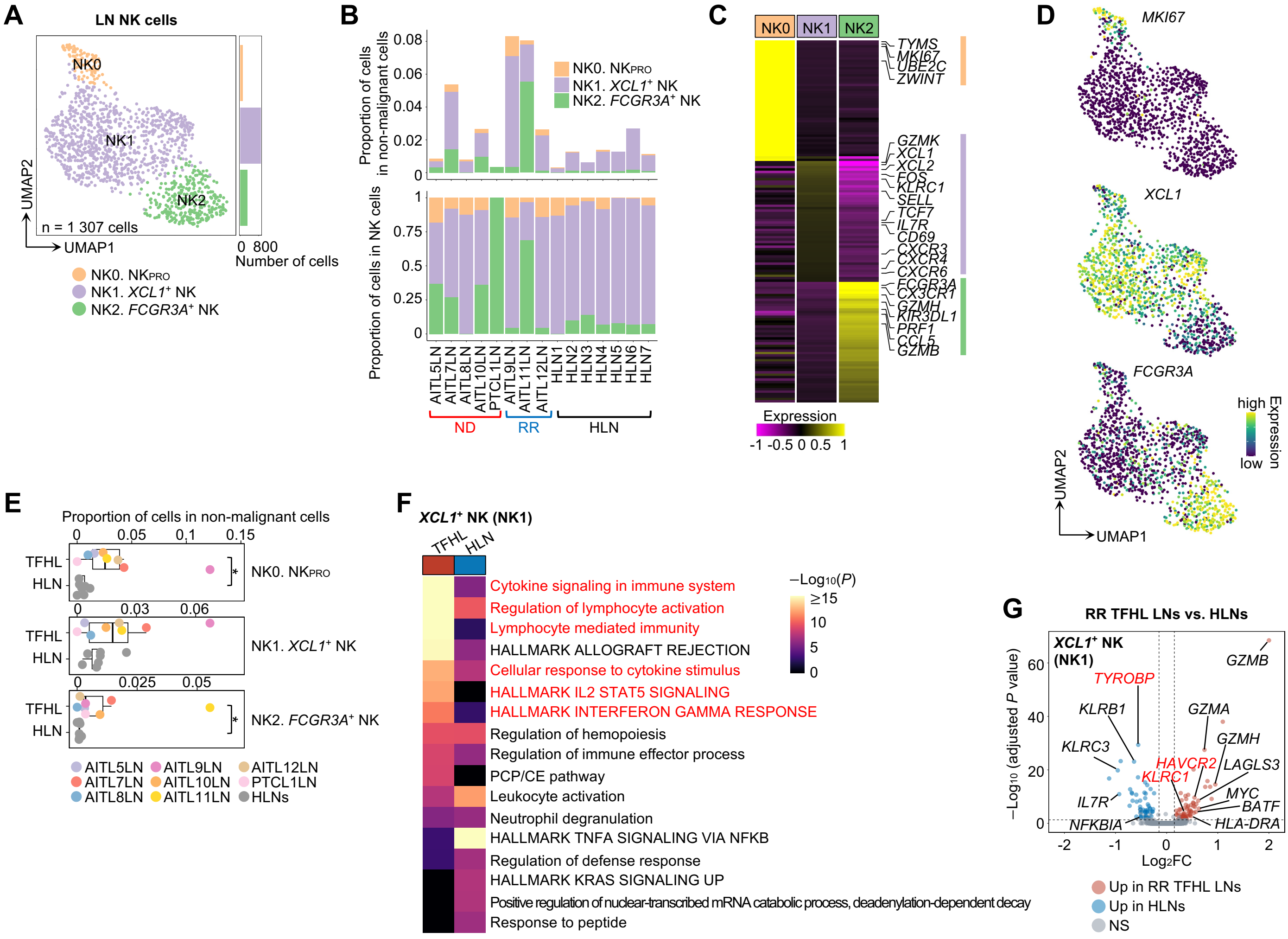

Figure S13. Subclustering of LN NK cells

(A) UMAP plot of LN NK-cell subclusters. *FCGR3A*$^{+}$ NK, *FCGR3A*-positive activated natural killer cell; NK_{PRO}, proliferative NK; *XCL1*$^{+}$ NK, *XCL1*-positive tissue-resident NK.

(B) Proportions of each LN NK-cell subcluster within non-malignant MNCs (top) and within NK cells (bottom) for each sample.

(C) Heatmap of the top 50 DEGs for each cluster of LN NK cells.

(D) Feature plots of marker genes in LN NK cells.

(E) Comparison of proportions of each subcluster of LN NK cells in non-malignant MNCs of each sample. The boxplots show the median (center line), interquartile range (box limits), minimum to max values (whiskers), and samples (dots) for each group. *P*-values are shown only when there is a significant difference. $*P < 5.0 \times 10^{-2}$.

(F) Heatmap of the top 10 pathways detected by GO analysis between *XCL1*$^{+}$ NKs (NK1) from TFHL LNs and those from HLNs.

(G) Volcano plot of DEGs in *XCL1*$^{+}$ NKs (NK1) from RR TFHL LNs (red) and those from HLNs (blue).

Supplementary Figure 14

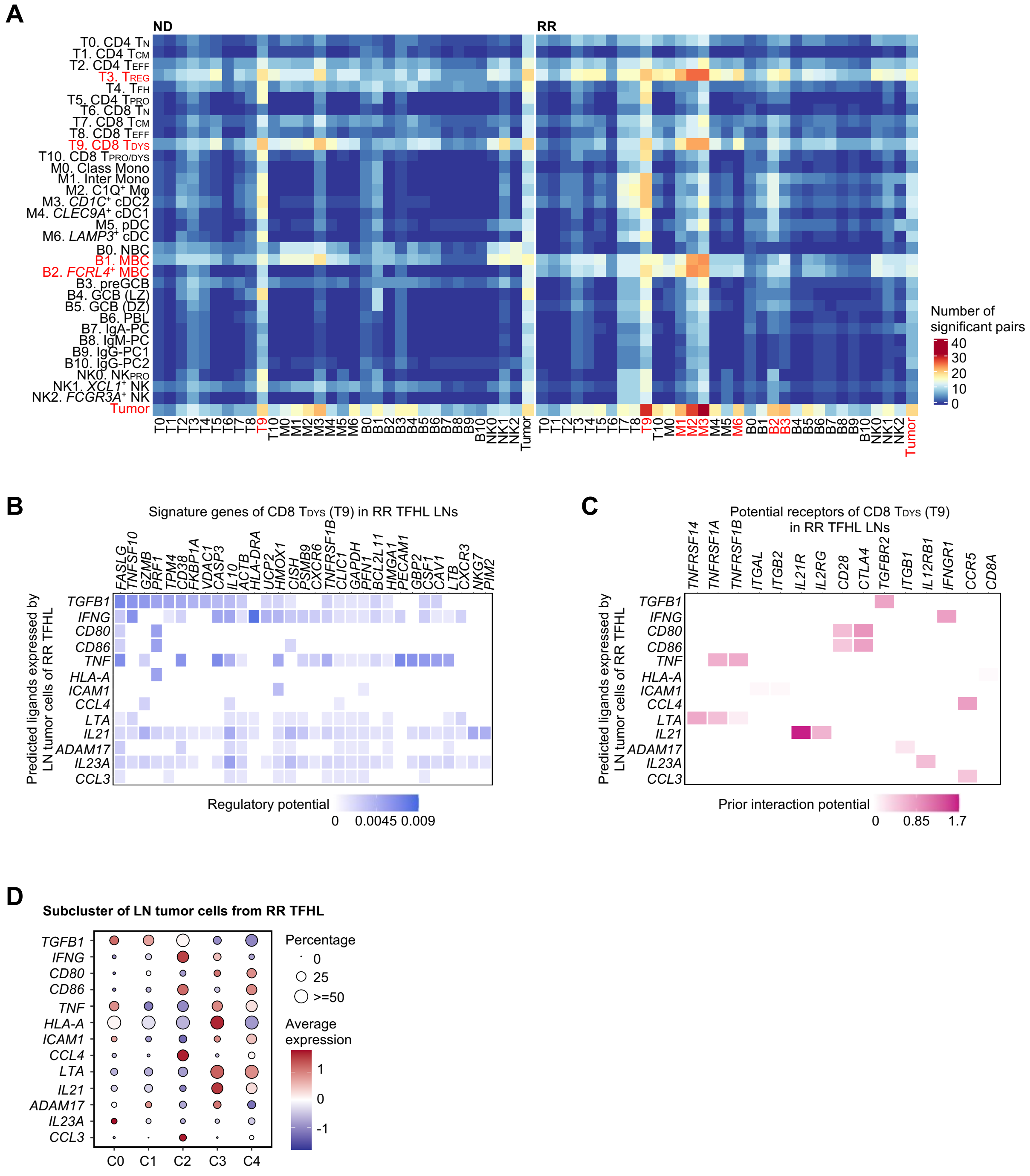

Figure S14. *In silico* cell-cell interaction analysis of TFHL LNs

(A) Number of significant interactions detected by *in silico* cellular interaction analysis using CellPhoneDB [29] performed between tumor cells and subclusters of immune cells from ND (left) and RR (right) TFHL LNs.

(B,C) Estimation of possible ligands (B), which can cause dysfunctional/exhausted signatures of CD8 T_{DYS} from RR TFHL LNs, and their receptors expressed on tumor cells (C) by NicheNetR [30]. Only interactions selected based on validated curated ligand-receptor databases are visualized.

(D) Dot plot of average expression per tumor-cell subcluster of the receptors that can drive the CD8 T_{DYS} signature estimated by NicheNetR.

Supplementary Figure 15

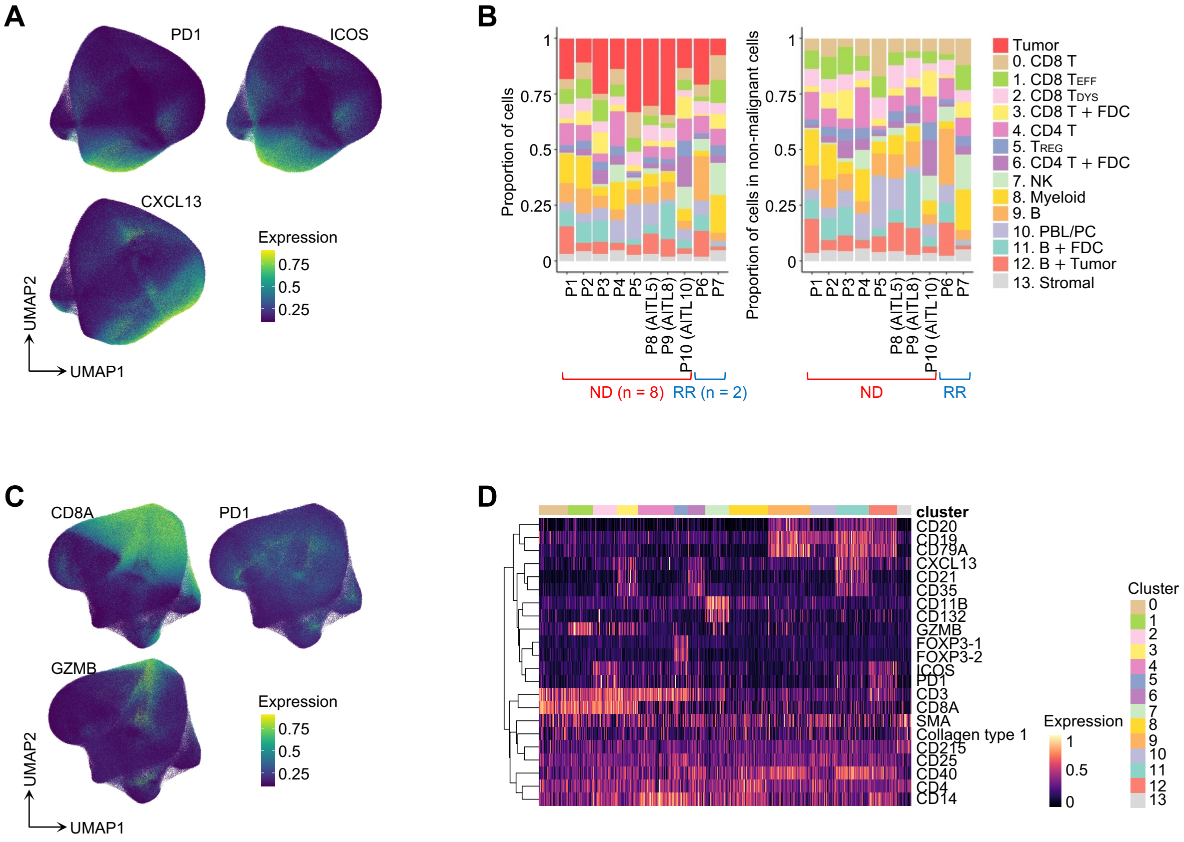

Figure S15. Single-cell spatial analysis of TFHL tissues

(A) Feature plots of T_{FH} markers in all cells from TFHL tissues.

(B) Proportions of each cluster within all cells (left) and within non-malignant cells (right) for each sample.

(C) Feature plots of markers related to $CD8^+$ T cell types in non-malignant cells.

(D) Heatmap of markers for each cluster of non-malignant cells.

Supplementary Figure 16

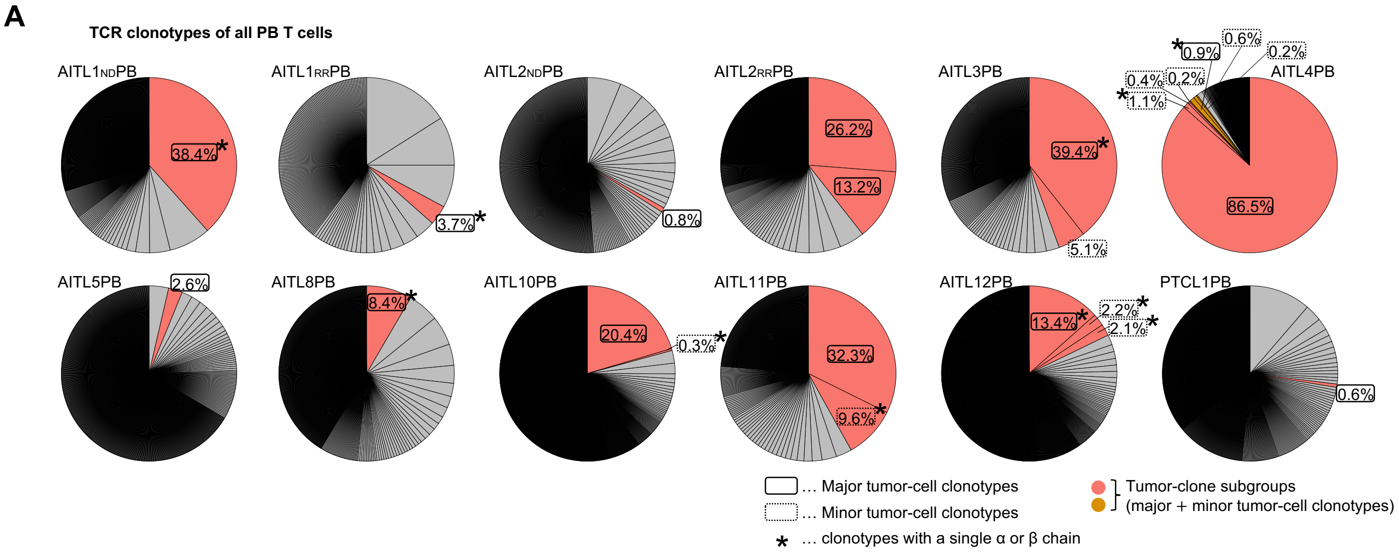

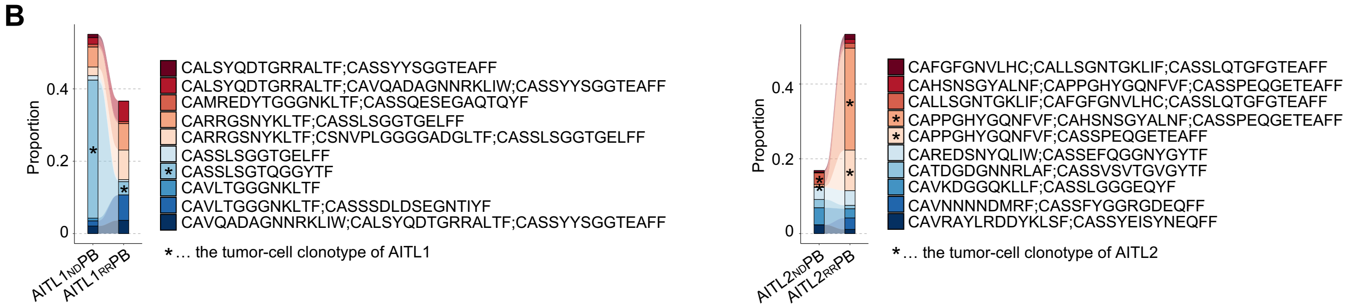

Figure S16. TCR repertoire analysis of PB samples

(A) Pie charts of TCR clonotypes for each sample. Tumor clones are colored. Major tumor-cell clonotypes are indicated by solid lines, and minor tumor-cell clonotypes are indicated by dotted lines. Clonotypes with only one TCR chain are indicated by an asterisk (*).

(B) Sankey plots showing TCR tracking of sequential samples (AITL1 and AITL2). The top 10 clonotypes of samples from ND (AITL1$_{ND}$ and AITL2$_{ND}$) and RR (AITL1$_{RR}$ and AITL2$_{RR}$) TFHLs were compared and CDR3 sequences of each clonotype are shown. The major clonotypes of tumor cells are indicated by an asterisk (*).

Supplementary Figure 17

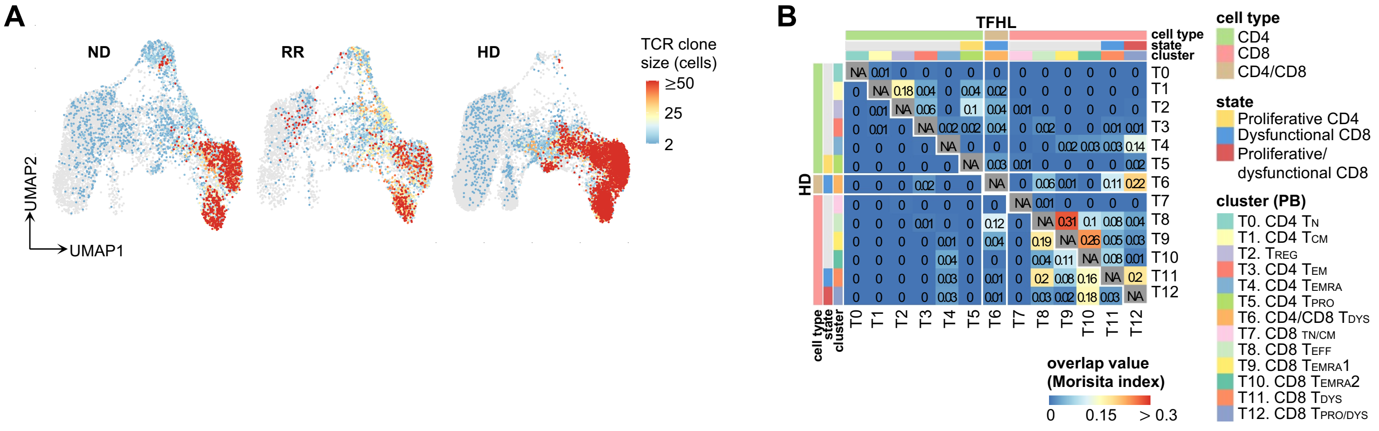

Figure S17. Characteristics of subclusters of non-malignant PB T cells

(A) Clone size of TCRs in non-malignant PB T cells. The number of cells expressing each clonotype was defined as clone size and illustrated for each cell.

(B) TCR overlap analysis of non-malignant PB T cells, analyzed and illustrated for PB from TFHL patients (upper right) and HDs (lower left), respectively.

Supplementary Figure 18

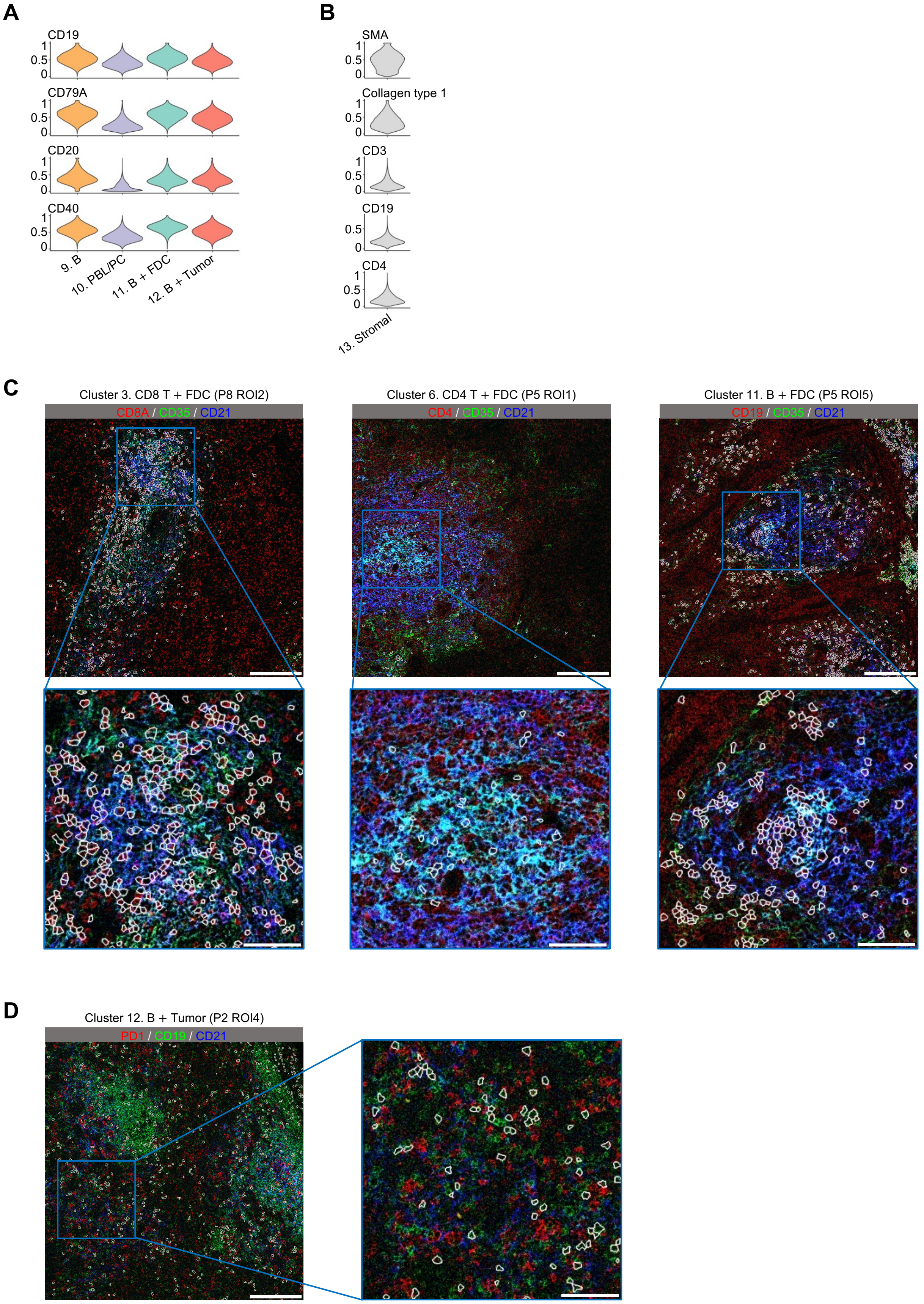

Figure S18. Annotation of subclusters of non-malignant cells in spatial analysis

(A) Violin plots of B-cell markers in B-cell clusters (clusters 9–12).

(B) Violin plots of stromal and lineage markers in a stromal-cell cluster (cluster 13).

(C) Representative IMC images of doublet clusters of FDCs and $CD8^+$ T (cluster 3, left), $CD4^+$ T (cluster 6, middle), and B (cluster 11, right) cells, colored according to expression levels of markers for FDCs (CD21 and CD35) and each cell (CD8A, CD4, and CD19, respectively). Cells belonging to each doublet cluster are outlined with white lines. ROI, region of interest; Scale bar, 300 μm (top) and 100 μm (bottom).

(D) Representative IMC images of doublet clusters of B and tumor cells (cluster 12) colored according to expression levels of markers for B cells (CD19), tumor cells (PD1), and FDCs (CD21). Cells belonging to each doublet cluster are outlined with white lines. Scale bar, 300 μm (left) and 100 μm (right).

Supplementary References

1 Fujisawa M, Nguyen TB, Abe Y, Suehara Y, Fukumoto K, Suma S *et al.* Clonal germinal center B cells function as a niche for T-cell lymphoma. *Blood* 2022; **140**: 1937–1950.

2 Wickham H. *ggplot2*. Springer International Publishing: Cham, 2016 doi:10.1007/978-3-319-24277-4.

3 Wen W, Su W, Tang H, Le W, Zhang X, Zheng Y *et al.* Immune cell profiling of COVID-19 patients in the recovery stage by single-cell sequencing. *Cell Discov* 2020; **6**: 31.

4 McCarthy DJ, Campbell KR, Lun ATL, Wills QF. Scater: pre-processing, quality control, normalization and visualization of single-cell RNA-seq data in R. *Bioinformatics* 2017; **33**: 1179–1186.

5 Villani A-C, Satija R, Reynolds G, Sarkizova S, Shekhar K, Fletcher J *et al.* Single-cell RNA-seq reveals new types of human blood dendritic cells, monocytes, and progenitors. *Science* 2017; **356**. doi:10.1126/science.aah4573.

6 Stuart T, Butler A, Hoffman P, Hafemeister C, Papalexi E, Mauck WM *et al.* Comprehensive Integration of Single-Cell Data. *Cell* 2019; **177**: 1888-1902.e21.

7 Haghverdi L, Lun ATL, Morgan MD, Marioni JC. Batch effects in single-cell RNA-sequencing data are corrected by matching mutual nearest neighbors. *Nat Biotechnol* 2018; **36**: 421–427.

8 Lavin Y, Kobayashi S, Leader A, Amir E-AD, Elefant N, Bigenwald C *et al.* Innate Immune Landscape in Early Lung Adenocarcinoma by Paired Single-Cell Analyses. *Cell* 2017; **169**: 750-765.e17.

9 Cheng S, Li Z, Gao R, Xing B, Gao Y, Yang Y *et al.* A pan-cancer single-cell

transcriptional atlas of tumor infiltrating myeloid cells. *Cell* 2021; **184**: 792-809.e23.

10 Yang C, Siebert JR, Burns R, Gerbec ZJ, Bonacci B, Rymaszewski A *et al.* Heterogeneity of human bone marrow and blood natural killer cells defined by single-cell transcriptome. *Nat Commun* 2019; **10**: 3931.

11 Pizzolato G, Kaminski H, Tosolini M, Franchini D-M, Pont F, Martins F *et al.* Single-cell RNA sequencing unveils the shared and the distinct cytotoxic hallmarks of human TCRVδ1 and TCRVδ2 γδ T lymphocytes. *Proc Natl Acad Sci U S A* 2019; **116**: 11906–11915.

12 Germain P-L, Lun A, Garcia Meixide C, Macnair W, Robinson MD. Doublet identification in single-cell sequencing data using scDblFinder. *F1000Research* 2021; **10**: 979.

13 Aran D, Looney AP, Liu L, Wu E, Fong V, Hsu A *et al.* Reference-based analysis of lung single-cell sequencing reveals a transitional profibrotic macrophage. *Nat Immunol* 2019; **20**: 163–172.

14 Padovan E, Casorati G, Dellabona P, Meyer S, Brockhaus M, Lanzavecchia A. Expression of two T cell receptor alpha chains: dual receptor T cells. *Science* 1993; **262**: 422–4.

15 Schuldt NJ, Binstadt BA. Dual TCR T Cells: Identity Crisis or Multitaskers? *J Immunol* 2019; **202**: 637–644.

16 Shi Z, Zhang Q, Yan H, Yang Y, Wang P, Zhang Y *et al.* More than one antibody of individual B cells revealed by single-cell immune profiling. *Cell Discov* 2019; **5**: 64.

17 Brady BL, Steinel NC, Bassing CH. Antigen receptor allelic exclusion: an update and reappraisal. *J Immunol* 2010; **185**: 3801–8.

18 ImmunoMind. immunarch: An R Package for Painless Bioinformatics Analysis of T-Cell

and B-Cell Immune Repertoires. Zenodo. 2019.

19 Finak G, McDavid A, Yajima M, Deng J, Gersuk V, Shalek AK *et al.* MAST: a flexible statistical framework for assessing transcriptional changes and characterizing heterogeneity in single-cell RNA sequencing data. *Genome Biol* 2015; **16**: 278.

20 Zhou Y, Zhou B, Pache L, Chang M, Khodabakhshi AH, Tanaseichuk O *et al.* Metascape provides a biologist-oriented resource for the analysis of systems-level datasets. *Nat Commun* 2019; **10**: 1523.

21 Subramanian A, Tamayo P, Mootha VK, Mukherjee S, Ebert BL, Gillette MA *et al.* Gene set enrichment analysis: a knowledge-based approach for interpreting genome-wide expression profiles. *Proc Natl Acad Sci U S A* 2005; **102**: 15545–50.

22 Hänzelmann S, Castelo R, Guinney J. GSVA: gene set variation analysis for microarray and RNA-seq data. *BMC Bioinformatics* 2013; **14**: 7.

23 Robinson MD, McCarthy DJ, Smyth GK. edgeR: a Bioconductor package for differential expression analysis of digital gene expression data. *Bioinformatics* 2010; **26**: 139–40.

24 McCarthy DJ, Chen Y, Smyth GK. Differential expression analysis of multifactor RNA-Seq experiments with respect to biological variation. *Nucleic Acids Res* 2012; **40**: 4288–97.

25 Kassambara A. rstatix:Pipe-Friendly Framework for Basic Statistical Tests. R package version 0.7.0. *https://CRANR-project.org/package=rstatix* 2021.

26 Qiu X, Hill A, Packer J, Lin D, Ma Y-A, Trapnell C. Single-cell mRNA quantification and differential analysis with Census. *Nat Methods* 2017; **14**: 309–315.

27 Cao J, Spielmann M, Qiu X, Huang X, Ibrahim DM, Hill AJ *et al.* The single-cell transcriptional landscape of mammalian organogenesis. *Nature* 2019; **566**: 496–502.

28 Street K, Risso D, Fletcher RB, Das D, Ngai J, Yosef N *et al.* Slingshot: cell lineage and pseudotime inference for single-cell transcriptomics. *BMC Genomics* 2018; **19**: 477.

29 Garcia-Alonso L, Handfield L-F, Roberts K, Nikolakopoulou K, Fernando RC, Gardner L *et al.* Mapping the temporal and spatial dynamics of the human endometrium in vivo and in vitro. *Nat Genet* 2021; **53**: 1698–1711.

30 Browaeys R, Saelens W, Saeys Y. NicheNet: modeling intercellular communication by linking ligands to target genes. *Nat Methods* 2020; **17**: 159–162.

31 Makishima K, Suehara Y, Abe Y, Hattori K, Kusakabe M, Matsuoka R *et al.* Intratumor heterogeneity of lymphoma identified by multiregion sequencing of autopsy samples. *Cancer Sci* 2022; **113**: 362–364.

32 Abe Y, Sakata-Yanagimoto M, Fujisawa M, Miyoshi H, Suehara Y, Hattori K *et al.* A single-cell atlas of non-haematopoietic cells in human lymph nodes and lymphoma reveals a landscape of stromal remodelling. *Nat Cell Biol* 2022; **24**: 565–578.

33 Robinson JT, Thorvaldsdóttir H, Winckler W, Guttman M, Lander ES, Getz G *et al.* Integrative genomics viewer. *Nat Biotechnol* 2011; **29**: 24–6.

34 McKenna A, Hanna M, Banks E, Sivachenko A, Cibulskis K, Kernytsky A *et al.* The Genome Analysis Toolkit: a MapReduce framework for analyzing next-generation DNA sequencing data. *Genome Res* 2010; **20**: 1297–303.

35 Petti AA, Williams SR, Miller CA, Fiddes IT, Srivatsan SN, Chen DY *et al.* A general approach for detecting expressed mutations in AML cells using single cell RNA-sequencing. *Nat Commun* 2019; **10**: 3660.

36 Tirosh I, Izar B, Prakadan SM, Wadsworth MH, Treacy D, Trombetta JJ *et al.* Dissecting the multicellular ecosystem of metastatic melanoma by single-cell RNA-seq. *Science* 2016; **352**: 189–96.

37 Kurtenbach S, Cruz AM, Rodriguez DA, Durante MA, Harbour JW. Uphyloplot2: visualizing phylogenetic trees from single-cell RNA-seq data. *BMC Genomics* 2021; **22**: 419.

38 Peng J, Sun B-F, Chen C-Y, Zhou J-Y, Chen Y-S, Chen H *et al.* Single-cell RNA-seq highlights intra-tumoral heterogeneity and malignant progression in pancreatic ductal adenocarcinoma. *Cell Res* 2019; **29**: 725–738.

39 Zanotelli VR, Bodenmiller B. ImcSegmentationPipeline: A pixel-classification based multiplexed image segmentation pipeline. *Zenodo* 2022. doi:10.5281/zenodo.3841961.

40 Berg S, Kutra D, Kroeger T, Straehle CN, Kausler BX, Haubold C *et al.* ilastik: interactive machine learning for (bio)image analysis. *Nat Methods* 2019; **16**: 1226–1232.

41 Stirling DR, Swain-Bowden MJ, Lucas AM, Carpenter AE, Cimini BA, Goodman A. CellProfiler 4: improvements in speed, utility and usability. *BMC Bioinformatics* 2021; **22**: 433.

42 Windhager J, Bodenmiller B, Eling N. An end-to-end workflow for multiplexed image processing and analysis. *bioRxiv* 2021.

43 Eling N, Damond N, Hoch T, Bodenmiller B. Cytomapper: an R/bioconductor package for visualisation of highly multiplexed imaging data. *Bioinformatics* 2020; **36**: 5706–8.

44 Sakaguchi S, Mikami N, Wing JB, Tanaka A, Ichiyama K, Ohkura N. Regulatory T Cells and Human Disease. *Annu Rev Immunol* 2020; **38**: 541–566.

45 Bagaev D V., Vroomans RMA, Samir J, Stervbo U, Rius C, Dolton G *et al.* VDJdb in 2019: database extension, new analysis infrastructure and a T-cell receptor motif compendium. *Nucleic Acids Res* 2020; **48**: D1057–D1062.

46 Swerdlow SH, Campo E, Pileri SA, Lee Harris N, Stein H, Siebert R *et al.* The 2016 revision of the World Health Organization classification of lymphoid neoplasms. Blood.

2016; **127**: 2375–2390.

47 Yabe M, Gao Q, Ozkaya N, Huet S, Lewis N, Pichardo JD *et al.* Bright PD-1 expression by flow cytometry is a powerful tool for diagnosis and monitoring of angioimmunoblastic T-cell lymphoma. *Blood Cancer J* 2020; **10**: 32.

48 Zhang L, Yu X, Zheng L, Zhang Y, Li Y, Fang Q *et al.* Lineage tracking reveals dynamic relationships of T cells in colorectal cancer. *Nature* 2018; **564**: 268–272.

49 Maier B, Leader AM, Chen ST, Tung N, Chang C, LeBerichel J *et al.* A conserved dendritic-cell regulatory program limits antitumour immunity. *Nature* 2020; **580**: 257–262.
